# TRAF2 and RIPK1 redundantly mediate classical NFκB signaling by TNFR1 and CD95-type death receptors

**DOI:** 10.1038/s41419-024-07325-x

**Published:** 2025-01-21

**Authors:** Jennifer Wagner, David Vredevoogd, Xin Yu, Dong Lu, Daniel S. Peeper, Heike M. Hermanns, Jin Wang, Harald Wajant, Daniela Siegmund

**Affiliations:** 1 Division of Molecular Internal Medicine, Department of Internal Medicine II, University Hospital Würzburg, Auvera Haus Grombühlstraße 12, 97080 Würzburg, Germany; 2 Division of Molecular Oncology and Immunology, Oncode Institute, The Netherlands Cancer Institute, Plesmanlaan 121, 1066 CX Amsterdam, the Netherlands; 3 The Verna and Marrs McLean Department of Biochemistry and Molecular Pharmacology, Baylor College of Medicine, Houston, TX 77030 USA; 4 Division of Hepatology, Department of Internal Medicine II, University Hospital Würzburg, Auvera Haus Grombühlstraße 12, 97080 Würzburg, Germany; 5 Department of Molecular and Cellular Biology, Baylor College of Medicine, Houston, TX 77030 USA; 6 Center for NextGen Therapeutics, Baylor College of Medicine, Houston, TX 77030 USA

**Keywords:** Tumour-necrosis factors, Apoptosis

## Abstract

This study suggests a modified model of TNFR1-induced complex I-mediated NFκB signaling. Evaluation of a panel of five tumor cell lines (HCT116-PIK3CAmut, SK-MEL-23, HeLa-RIPK3, HT29, D10) with TRAF2 knockout revealed in two cell lines (HT29, HeLa-RIPK3) a sensitizing effect for death receptor-induced necroptosis and in one cell line (D10) a mild sensitization for TNFR1-induced apoptosis. TRAF2 deficiency inhibited death receptor-induced classical NFκB-mediated production of IL-8 only in a subset of cell lines and only partly. TRAF5, furthermore, failed to improve DR-induced NFκB signaling in HCT116-PIK3CAmut and HCT116-PIK3CAmut-TRAF2_KO_ cells. These findings argue for a non-obligatory role of TRAF2 in death receptor-induced classical NFκB signaling. Similar as in TRAF2-deficient cells, TNF- and CD95L-induced NFκB signaling was found to be only poorly affected in RIPK1_KO_ cells and in cells treated with the RIPK1-specific PROTAC LD4172. Intriguingly, however, death receptor-induced NFκB signaling was completely inhibited in HCT116-PIK3CAmut cells double deficient for TRAF2 and RIPK1 and in TRAF2-deficient cells treated with LD4172. Moreover, with exception of recruitment of TRADD, acting upstream to TRAF2 and parallel to RIPK1, TNFR1 signaling complex formation was abrogated in TRAF2-RIPK1 DKO cells. Based on our findings, two distinguishable types of TNFR1-interacting complexes promote TNF-induced NFκB signaling: First, a TRADD-TRAF2/cIAP utilizing complex Ia which becomes evident in RIPK1-deficient cells. Second, a non-modified RIPK1 utilizing complex Ib which acts in TRADD- or TRAF2-deficient cells. Complex Ia and Ib may furthermore interact and cooperate to ubiquitinate RIPK1 resulting in a modified complex Ia/b preventing complex Ia and Ib to convert to the established TNFR1-induced cytotoxic complexes IIa and IIb.

## Introduction

The death receptor (DR) subgroup of the tumor necrosis factor (TNF) receptor superfamily (TNFRSF) is characterized by a conserved cytoplasmic protein-protein interaction domain, the name giving death domain (DD) [1]. The death receptors can be further subdivided with respect to their interaction partners and signaling mechanisms [1, 2]: CD95 (Apo1/Fas) and the TRAIL death receptors TRAILR1 (DR4) and TRAILR2 (DR5) interact by virtue of their DD with the DD of the adapter protein Fas-associated death domain (FADD) which mediates recruitment of procaspase-8 and its regulatory homologs FLIP_S_, FLIP_L_ and FLIP_R_ [3]. Death receptor-associated procaspase-8 can furthermore recruit additional procaspase-8 or FLIP molecules by homotypic interaction of death effector domains (DEDs) which are present at the N-terminus of procaspase-8 and the FLIP proteins and which are structurally related to the DD [3]. Dependent on the ratio of procaspase-8 and FLIP proteins in the receptor signaling complexes of CD95 and the TRAIL death receptors, autoproteolytic processing of procaspase-8 and release of mature caspase-8 is possible which can eventually trigger apoptotic cell death [3]. TNFR1 and DR3 do not directly interact with FADD and thus do not recruit procaspase-8 [1, 2]. Instead, these death receptors interact by DD-DD interactions with the adapter protein TRADD and the receptor interacting protein kinase-1 (RIPK1) molecule. Nevertheless, TNFR1 and DR3 can instruct, in a TRADD/RIPK1-dependent fashion, the formation of cytoplasmic FADD and procaspase-8-containing protein complexes to produce mature caspase-8 [2, 4]. The remaining DRs, p75NGFR, EDAR and DR6, only induce cell death under poorly defined circumstances without a clear evidence for an involvement of the FADD/procaspase-8 axis. The CD95- (CD95, TRAILR1, TRAILR2) and TNFR1-type (TNFR1, DR3) DRs are also able to trigger necroptotic cell death via a RIPK1-RIPK3 axis. Intriguingly, however, FADD is here of opposite relevance. It is essentially required for necroptosis induction by CD95 and the TRAIL death receptors, but dispensable and even inhibitory in TNFR1-induced necroptosis [5, 6].

Despite their name, DRs are not only able to trigger cell death programs but can also stimulate various cell death-independent signaling pathways, especially the strongly proinflammatory classical nuclear factor of kappaB (NFκB) pathway. It is worth mentioning that the aforementioned cell death signaling proteins FADD, TRADD, RIPK1 have also been implicated in classical NFκB signaling by the CD95- and TNFR1-type death receptors but again in a receptor-specific fashion. While FADD seems to be required for the activation of the classical NFκB pathway by the CD95-type DRs, it is dispensable for TNFR1-dependent NFκB signaling [2]. TRADD, RIPK1 and their interaction partner TNF receptor-associated factor-2 (TRAF2), however, have been implicated in NFκB signaling by both types of death receptors in a non-obligatory fashion [2]. Especially in the context of TNFR1 signaling, it has been suggested that RIPK1 and TRAF2 cooperate in a sequential, thus hierarchic manner in NFκB signaling. TRAF2-associated cellular inhibitor of apoptosis (cIAP) molecules (cIAP1 or cIAP2) K63-ubiquitinate RIPK1 to create docking sites for the linear ubiquitin chain assembly complex (LUBAC). The latter promotes then linear ubiquitination of TNFR1 signaling complex components resulting in docking sites for the inhibitor of κB kinase (IKK) complex-stimulating TAB2-TAK1 complex and the IKK complex enabling phosphorylation and subsequent degradation of IκB proteins, the central event in the classical NFκB pathway [7]. This hierarchic mode of action of RIPK1 and TRAF2 in NFκB signaling has not yet been evaluated for the CD95-type of death receptors and is challenged for TNFR1 signaling by the fact that TRAF2 and RIPK1 knockout cells often show considerable residual NFκB signaling. Here, we demonstrate that TRAF2-RIPK1 double deficiency results in a strong and comprehensive abrogation of DR-induced NFκB signaling while corresponding single TRAF2 or RIPK1 knockout cells are only partly affected. This suggests that beyond the hierarchic and cooperative mode of NFκB signaling described above each of the two components has an own and independent capacity to transduce DR-induced NFκB-stimulating signals.

## Results

### TRAF2 protects cells to a variable extent from TNFR1- and CD95 death receptor type induced cell death

**Fig. 1:**
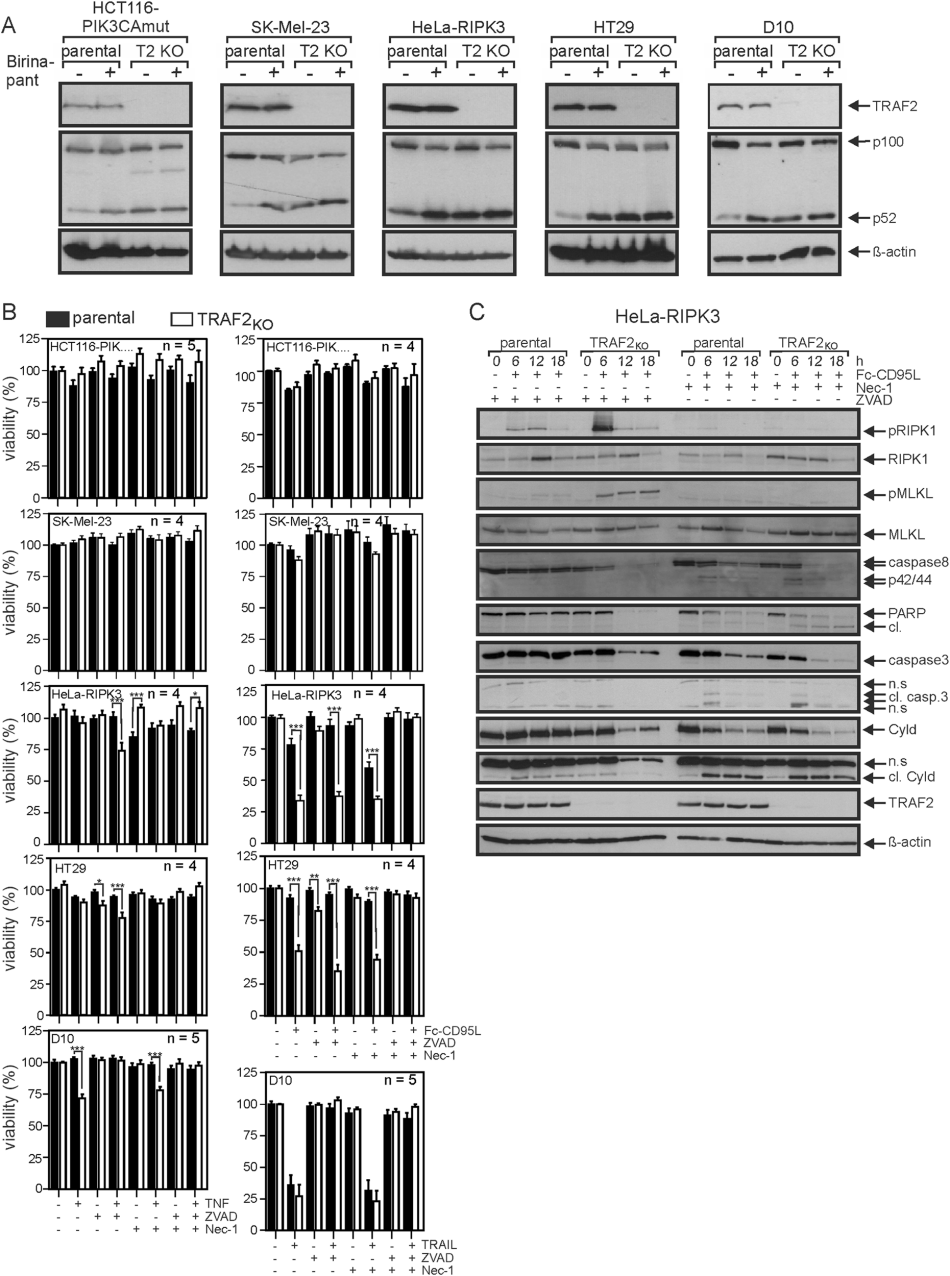
TRAF2 deficiency sensitizes in a cell type-specific manner for death receptor-induced cell death. **A** The indicated cell lines and their corresponding TRAF2-deficient variants were stimulated for 18 h with birinapant (1 µM) and total cell lysates were analyzed for p100 processing to p52. One of two or three experiments with similar results are shown. **B** Cells were stimulated overnight as indicated with 100 ng/ml TNF, 200 ng/ml Fc-CD95L, 200 ng/ml TRAIL, 20 µM ZVAD or 90 µM necrostatin-1 (Nec-1). After 16–18 h, cell viability was evaluated by crystal violet staining. Shown are the mean +/− SEM of 4 or 5 independent experiments. Cell death induction was statistically evaluated by two-way ANOVA of parental and TRAF2-deficient cells. ***p < 0.001; **p < 0.01; *p < 0.05. **C** HeLa-RIPK3 cells along with their TRAF2-deficient counterparts were stimulated for 6, 12 and 18 h with Fc-CD95L (200 ng/ml) plus ZVAD (20 µM) or Fc-CD95L plus Nec-1 (90 µM) or remained untreated. Total cell lysates were evaluated by Western blot with respect to the expression/phosphorylation of the indicated proteins.

Initially, we analyzed a panel of TRAF2-deficient human cell lines with respect to TNF- and CD95L-induced cytotoxicity. The cell line panel was compiled with the aim of including cell lines with and without necroptosis competence and different apoptosis sensitivity. TNF and CD95L were further chosen as stimuli to cover the TNFR1- and CD95-like class of death receptors. HT29 cells and HeLa cells stably transfected with RIPK3 (HeLa-RIPK3) are two of the most intensively studied cell variants regarding death receptor-induced necroptosis. HCT116-PIK3CAmut cells are an isogenic cell line variant of HCT116 cells expressing only a mutated active *PIK3CA* oncogene allele which drives constitutive Bax degradation so that death receptor-induced apoptosis occurs exclusively via the extrinsic pathway without cooperation with the intrinsic apoptosis pathway [8, 9]. D10 and SK-Mel-23 were furthermore included due to the fact that TRAF2-deficient variants were already available from previous studies and in the case of D10 have documented sensitivity for TNF-induced apoptosis [10]. A biochemical hallmark of TRAF2 deficiency, but also of deficiency for TRAF3 as well as cIAP1 and cIAP2, is enhanced processing of the NFκB precursor protein p100 to p52. This reflects the crucial relevance of these four proteins for the constitutive inhibition of the alternative NFκB pathway [11–13]. Indeed, western blot analysis revealed enhanced p100 processing in all five TRAF2-deficient cell lines (Fig. 1A). Moreover, the extent of p100 to p52 processing observed in the TRAF2-deficient cells was largely comparable to those induced in the parental cell line variants by birinapant, an effective inhibitor of cIAP1, cIAP2 and xIAP (Fig. 1A) [14]. In accordance with our previous work [15], we found furthermore that TRAF2 deficiency does not break the resistance against extrinsic apoptosis of HCT116-PIK3CAmut cells (Fig. 1B). There was also no necroptosis induction in the HCT116-PIK3CAmut-TRAF2_KO_ cells as viability of TNF- or CD95L-treated cells remained unchanged even in the presence of ZVAD which can sensitize for necroptosis induction (Fig. 1B). Similarly, TNF and CD95L failed to trigger cell death in SK-MEL-23 and TRAF2-deficient SK-MEL-23 cells (Fig. 1B). HeLa-RIPK3 and HT29 turned out to be TNF resistant and only the HeLa-RIPK3 cells showed moderate sensitivity for CD95L-induced cell death (Fig. 1B, C). TRAF2-deficient HeLa-RIPK3 and HT29 cells, however, showed significantly enhanced CD95L-induced cell death (Fig. 1B). CD95L-induced cell death was still enhanced in the TRAF2-deficient cells in the presence of the pan-caspase inhibitor ZVAD as well as in the presence of the RIPK1 inhibitor Nec-1 (Fig. 1B, C). Combined treatment with ZVAD and Nec-1, however, rescued cells from death-induction (Fig. 1B). TRAF2-deficient HeLa-RIPK3 cells were also weakly sensitized for TNF-induced necroptosis (Fig. 1B). TRAF2-deficient D10 cells showed enhanced TNF-induced apoptosis (Fig. 1B). There was no apoptotic activity of CD95L on D10 cells even upon sensitization with CHX (Supplementary Fig. S1B). Flow cytometry, however, revealed poor CD95 expression in D10 cells (Supplementary Fig. S1B). We therefore analyzed D10 cells also for cell death induction by TRAIL which stimulates the CD95-type death receptors TRAILR1 and TRAILR2. Indeed, TRAIL efficiently induced apoptosis in D10 cells, but this response was not significantly enhanced by TRAF2 deficiency (Fig. 1B). In sum, the data derived from our panel of TRAF2-deficient cell lines indicate that TRAF2 has anti-apoptotic and anti-necroptotic activities in cell death-induction by both TNFR1- (Fig. 1B, left column) and CD95-type (Fig. 1B, right column) death receptors. Similar survival activities have been previously reported by us and others in HaCaT, L929 and HT-22 cells with TRAF2 knockdown and cardiomyocytes derived of TRAF2 knockout mice [16–19].

### TRAF2 and RIPK1 are not essential for TNFR1 and CD95-induced classical NFκB signaling

**Fig. 2:**
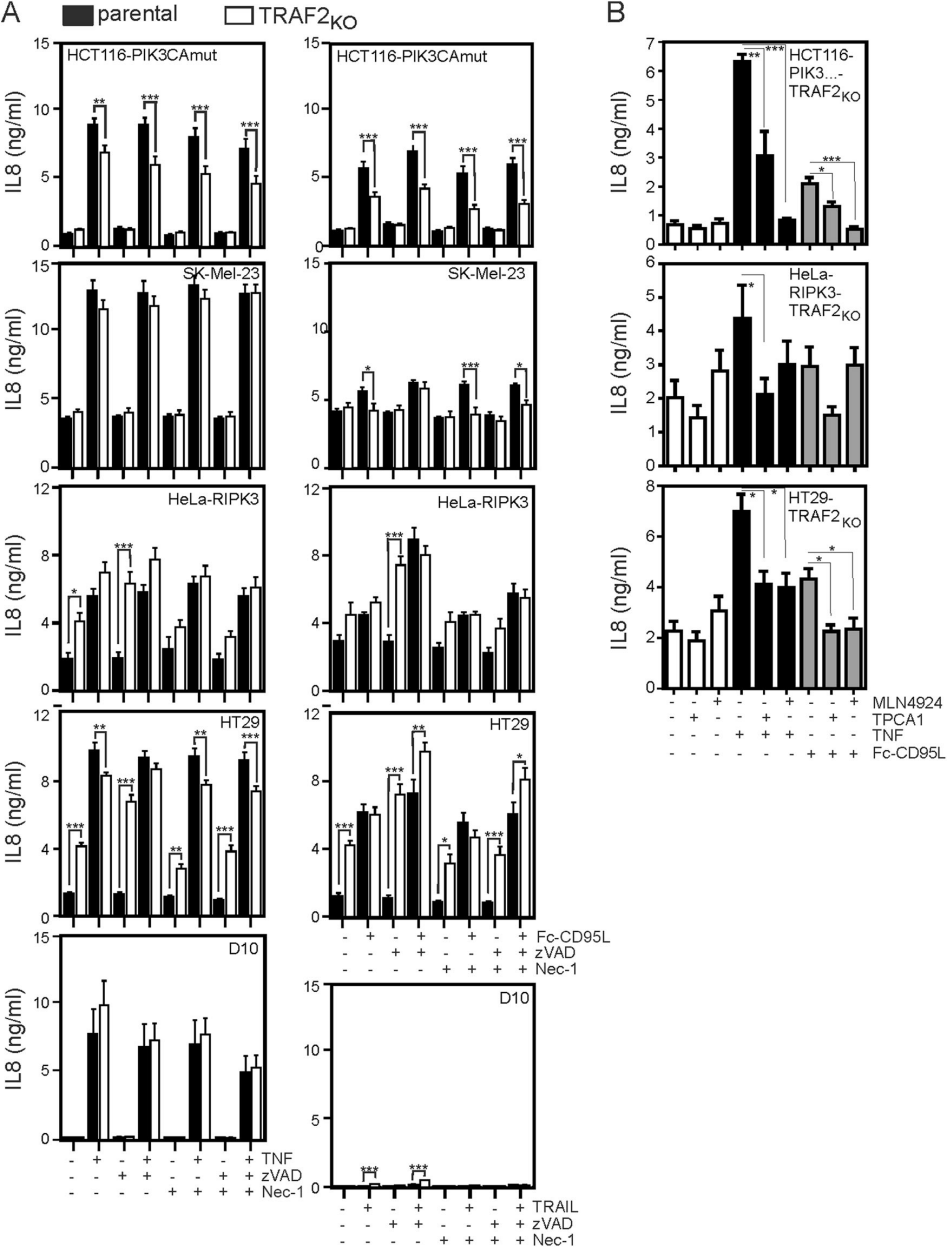
TRAF2 is largely dispensable for death receptor-induced NFκB-mediated IL-8 production. **A** Cells were stimulated as indicated with 100 ng/ml TNF, 200 ng/ml Fc-CD95L, 200 ng/ml TRAIL, 20 µM ZVAD or 90 µM necrostatin-1 (Nec-1). After 16–18 h, IL-8 production was quantified by ELISA analysis of cell culture supernatants. Shown are the mean +/− SEM of 4 independent experiments. IL-8 production was statistically evaluated by two-way ANOVA of parental and TRAF2-deficient cells. ***p < 0.001; **p < 0.01; *p < 0.05. **B** Cells were treated as indicated with 100 ng/ml TNF, 200 ng/ml Fc-CD95L, 20 µM TPCA-1 or 20 µM MLN4924. After 16–18 h, IL-8 production was quantified by ELISA analysis of cell culture supernatants. Shown are the mean +/− SEM of 4 independent experiments. IL-8 production data sets of untreated, TPCA-1 and MLN4923 were statistically evaluated by one-way ANOVA (Bonferroni post-hoc test). ***p < 0.001; **p < 0.01; *p < 0.05.

In a previous study, we found that induction of the expression of the classical NFκB pathway target protein IL-8 by TNFR1 and death receptors of the CD95 type is significantly reduced in TRAF2-deficient HCT116-PIK3CAmut cells [15]. To reinforce this initial finding, we evaluated the ability of TNF and CD95L to trigger IL-8 production in the TRAF2-deficient cell line variants shown in Fig. 1. As reported before, there was a significant reduction in the IL-8 production induced by TNF and Fc-CD95L in the HCT116-PIK3CAmut-TRAF2_KO_ cells (Fig. 2A). The weak CD95L-induced IL-8 production was abrogated in the SK-Mel-23-TRAF2_KO_ cells, but there was no significant reduction in the strong TNF-induced IL-8 production (Fig. 2A). In D10 cells, TNF induced a strong IL-8 production which, similarly to SK-Mel-23 cells, remained again unaffected by TRAF2 deficiency. Stimulation with TRAIL showed almost no IL-8 inducing capacity in this cell line irrespective of TRAF2 expression (Fig. 2A).

The situation in the HT29 and HeLa-RIPK3 variants, however, was more complex. Both, TNF and CD95L, induced significant IL-8 production in the parental HT29 and HeLa-RIPK3 cells and simultaneous application of ZVAD slightly enhanced this response in the CD95L-treated HeLa-RIPK3 cells which are mildly sensitive for CD95L-induced apoptosis (Fig. 1A, B). Intriguingly, in HT29-TRAF2_KO_ and HeLa-RIPK3-TRAF2_KO_ cells, there was already a robustly enhanced IL-8 production in the absence of exogenous receptor stimulation and this constitutively enhanced IL-8 secretion was further enhanced by the sole treatment with ZVAD (Fig. 2A). In contrast, in the parental HT29 and HeLa-RIPK3 cells ZVAD had no effect on basal IL-8 production (Fig. 2A). Moreover, TRAF2 deficiency and ZVAD treatment had no effect on the basal production of IL-8 in the HCT116-PIK3CAmut, SK-MEL-23 and D10 cells. Thus, IL-8 induction by TRAF2 deficiency and ZVAD application correlated with the necroptosis competence of the cell lines. Consequently, the RIPK1 inhibitor Nec-1 strongly reduced the ZVAD-induced increase in IL-8 production in HT29-TRAF2_KO_ and HeLa-RIPK3-TRAF2_KO_ cells (Fig. 2A). TNF stimulated additional IL-8 production in HT29-TRAF2_KO_ and HeLa-RIPK3-TRAF2_KO_ but in the presence of ZVAD this induction was not higher despite the IL-8 production-stimulatory effect of ZVAD (Fig. 2A). Similarly, CD95L induced no (HeLa-RIPK3-TRAF2_KO_) or only modest (HT29-TRAF2_KO_) IL-8 production in the TRAF2-deficient variants (Fig. 2A). Likewise, ZVAD-induced IL-8 production seemed to be not or only poorly increased by CD95 stimulation (Fig. 2A). In sum, under consideration that IL-8 production is constitutively higher and also inducible by ZVAD alone in HT29-TRAF2_KO_ and HeLa-RIPK3-TRAF2_KO_ cells, it appears that TNF and CD95L have a reduced ability to stimulate IL-8 production in these cell variants.

The classical NFκB pathway plays a major and crucial role in the induction of IL-8 production by cytokines but other pathways, e.g. the various MAP kinase cascades are also important [20]. To control that the considerable remaining TNF- and CD95L-induced IL-8 production observed in HCT116-PIK3CAmut-TRAF2_KO_, HT29-TRAF2_KO_ and HeLa-RIPK3-TRAF2_KO_ cells indeed still rely on activation of the classical NFκB pathway, we challenged the cells with TNF and Fc-CD95L in the presence of TPCA-1 or MLN4924. These two compounds inhibit stimulation of the classical NFκB pathway by different mechanisms, namely either by inhibition of the IκBα phosphorylating IKK2 subunit of the IKK complex (TPCA-1) or by inhibition of the IκBα K48 ubiquitinating E3 ligase Skp1-Cullin-F box (MLN4924). TPCA-1 showed a mild inhibitory effect on basal IL-8 production, but inhibited TNF- and CD95L-induced IL-8 production in all tested TRAF2_KO_ cell lines by approx. 50% (Fig. 2B). MLN4924, in contrast to TPCA-1, caused a minor increase in basal IL-8 production but nevertheless inhibited TNF- and Fc-CD95L-induced IL-8 production in HCT116-PIK3CAmut-TRAF2_KO_ and HT29-TRAF2_KO_, while the effect on HeLa-RIPK3-TRAF2_KO_ was less pronounced for TNF and absent for Fc-CD95L (Fig. 2B). Together, these results indicate that death receptor-induced IL-8 production in TRAF2-deficient cells indeed still involves the classical NFκB pathway.

**Fig. 3:**
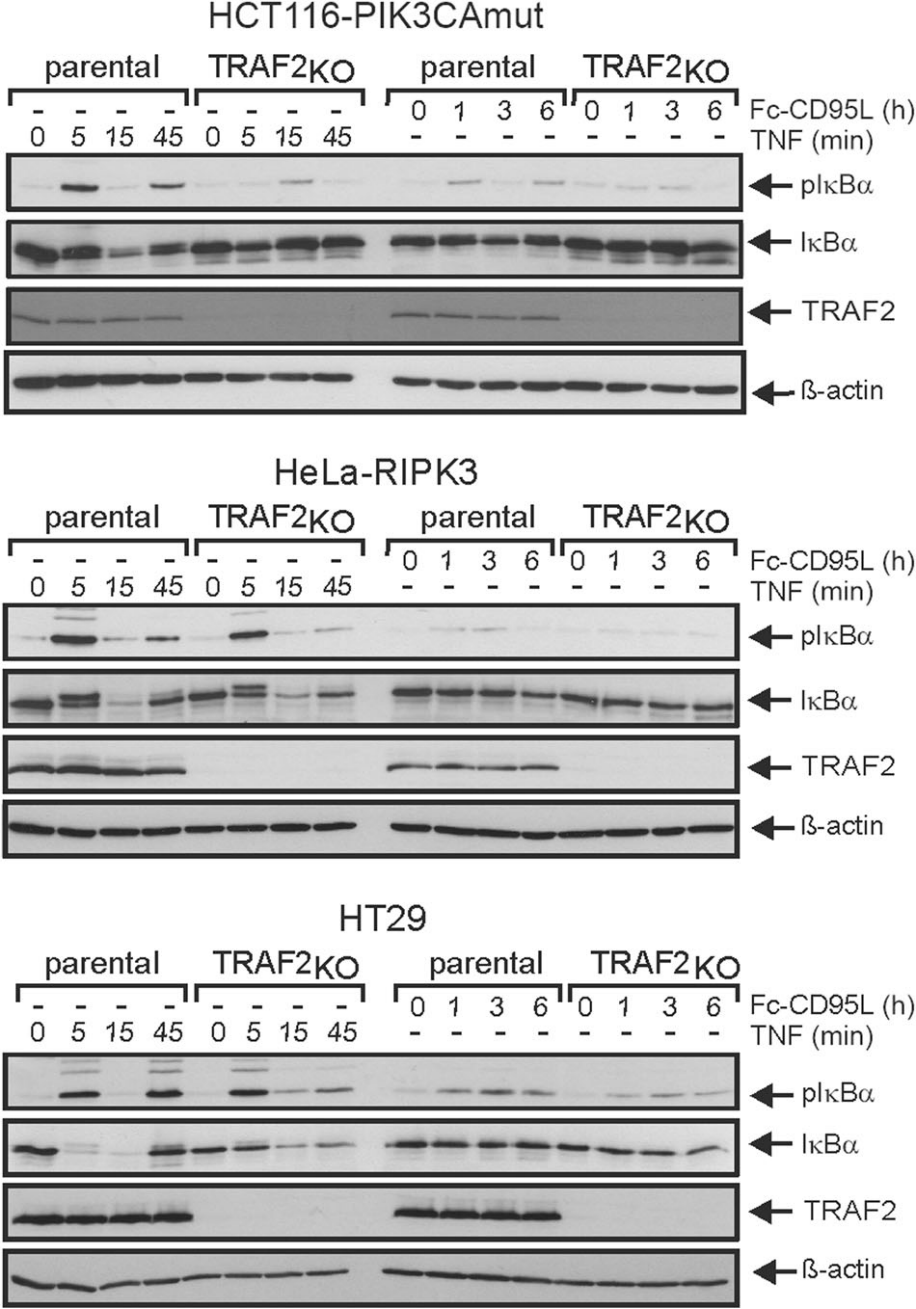
TRAF2 is largely dispensable for death receptor-induced NFκB signaling. The indicated parental cell lines along with their TRAF2-deficient counterparts were stimulated for the indicated times with 100 ng/ml TNF or 200 ng/ml Fc-CD95L. Total cell lysates were then evaluated with respect to the presence of pIκBα, IκBα and TRAF2 by western blotting.

In three of the matched parental and TRAF2-deficient cell line pairs, we also analyzed phosphorylation and degradation of IκBα as biochemical hallmark of activation of the classical NFκB pathway. In the parental TNF-treated cell line variants, IκBα was rapidly phosphorylated within 5 min, largely disappeared after 15 min and showed reappearance and phosphorylation after 45 min (Fig. 3, left panel). This is in good accordance with the known kinetics of phosphorylation, proteasomal degradation and NFκB-induced resynthesis of IκBα [11]. In the TRAF2-deficient cell variants of HCT116-PIK3CAmut and HT29 these TNF-induced events were reduced, but not completely abolished (Fig. 3). In HeLa-RIPK3 cells TRAF2-deficiency showed even no obvious effect on the levels of IκBα and phospho-IκBα (Fig. 3). CD95L-induced phosphorylation and degradation of IκBα occurred with a much slower kinetic and were significantly weaker than in response to TNF what is in accordance with earlier reports (e.g. ref. [21]). Again in the TRAF2-deficient cells, these responses were attenuated (Fig. 3). Taken together, these data suggest that TRAF2 contributes to a varying degree to death receptor-induced activation of the classical NFκB pathway, however, without being essential.

### TRAF2 and RIPK1 can independently mediate activation of the classical NFκB pathway by TNFR1 and death receptors of the CD95 type

**Fig. 4:**
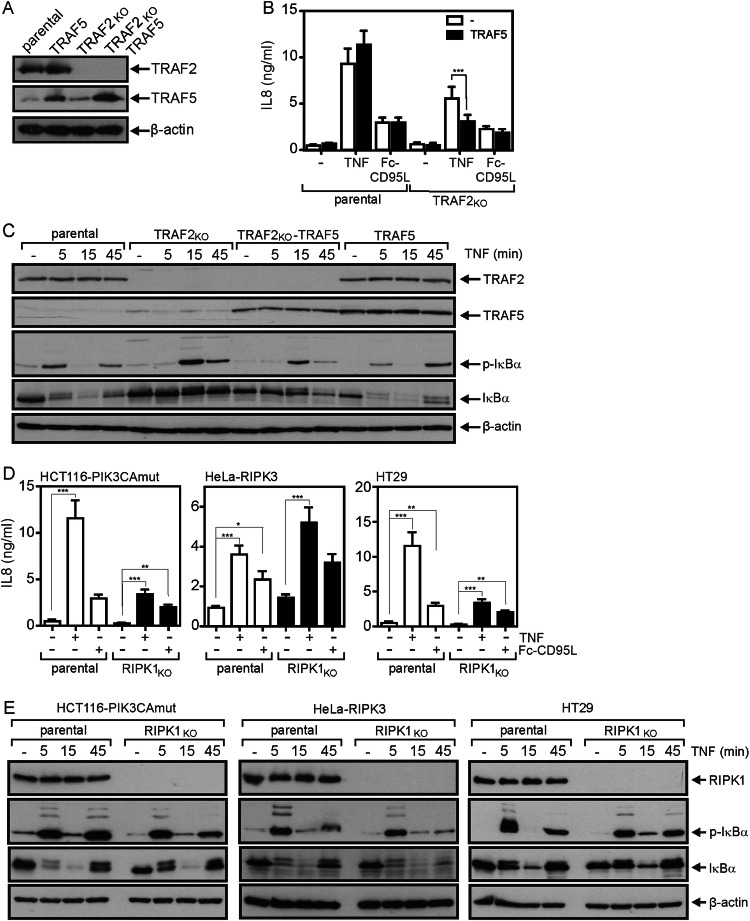
TRAF5 and RIPK1 have no major effect on death receptor-induced IL-8 induction. **A** Expression of TRAF2 and TRAF5 in HCT116-PIK3CAmut, HCT116-PIK3CAmut-TRAF5, HCT116-PIK3CAmut-TRAF2_KO_ and HCT116-PIK3CAmut-TRAF2_KO_-TRAF5 cells. **B** IL-8 secretion after overnight stimulation with TNF (100 ng/ml) or Fc-CD95L (200 ng/ml) of cell line variants shown in **A**. Data shown are mean +/− SEM of six independent experiments. IL-8 production was statistically evaluated in similarly treated pairs of cells transfected or not with TRAF5 by t-test. ***p < 0.001. **C** The indicated HCT116-PIK3CAmut variants were stimulated with 100 ng/ml TNF and total cell lysates were analyzed by western blotting with respect to the indicated protein species. One of two experiments with similar result is shown. **D** RIPK1-deficient variants of HCT116-PIK3CAmut, HeLa-RIPK3 and HT29 cells along with their parental counterparts were analyzed for DR-induced IL-8 production as described in **B**, n = 6. IL-8 production was statistically evaluated by one-way ANOVA (Bonferroni post-hoc test) *p < 0.05, **p < 0.01. ***p < 0.001. **E** Western blot analysis of phosphorylation and degradation of IκBα upon stimulation with TNF (100 ng/ml). One of two experiments with similar result is shown.

Since it has been reported that TRAF5 can substitute for TRAF2 in TNF-induced NFκB signaling [22], we stably transfected HCT116-PIK3CAmut and HCT116-PIK3CAmut- TRAF2_KO_ cells with TRAF5 and then analyzed death receptor-induced IL-8 production. HCT116-PIK3CAmut and HCT116-PIK3CAmut-TRAF2_KO_ cells showed low, but significant and comparable expression of endogenous TRAF5 (Fig. 4A). Using a TRAF5 encoding sleeping beauty expression vector, we obtained stable TRAF5 transfectants of HCT116-PIK3CAmut and HCT116-PIK3CAmut-TRAF2_KO_ cells with increased and comparable expression levels (Fig. 4A). Despite their enhanced TRAF5 expression levels, neither HCT116-PIK3CAmut-TRAF5 nor HCT116-PIK3CAmut-TRAF2_KO_-TRAF5 cells showed an increase in constitutive or TNF- and CD95L-induced IL-8 production (Fig. 4B). On the contrary, in the TRAF2_KO_ cells with ectopic TRAF5 expression there was even a significant reduction in TNF-induced IL-8 production (Fig. 4B). Likewise, TNF-induced phosphorylation and degradation of IκBα remained largely unaffected by increased TRAF5 expression (Fig. 4C). Intriguingly, IL-8 induction by TWEAK, which stimulates the TRAF5 utilizing TNFRSF member Fn14 [23], was clearly increased in HCT116-PIK3CAmut-TRAF5 and HCT116-PIK3CAmut-TRAF2_KO_-TRAF5 cells indicating that the elevated TRAF5 levels in the transfectants are of functional relevance (Supplementary Fig. S2). Thus, at least in HCT116-PIK3CAmut cells, we found no evidence for a redundant role of TRAF2 and TRAF5 in death receptor-induced NFκB signaling.

**Fig. 5:**
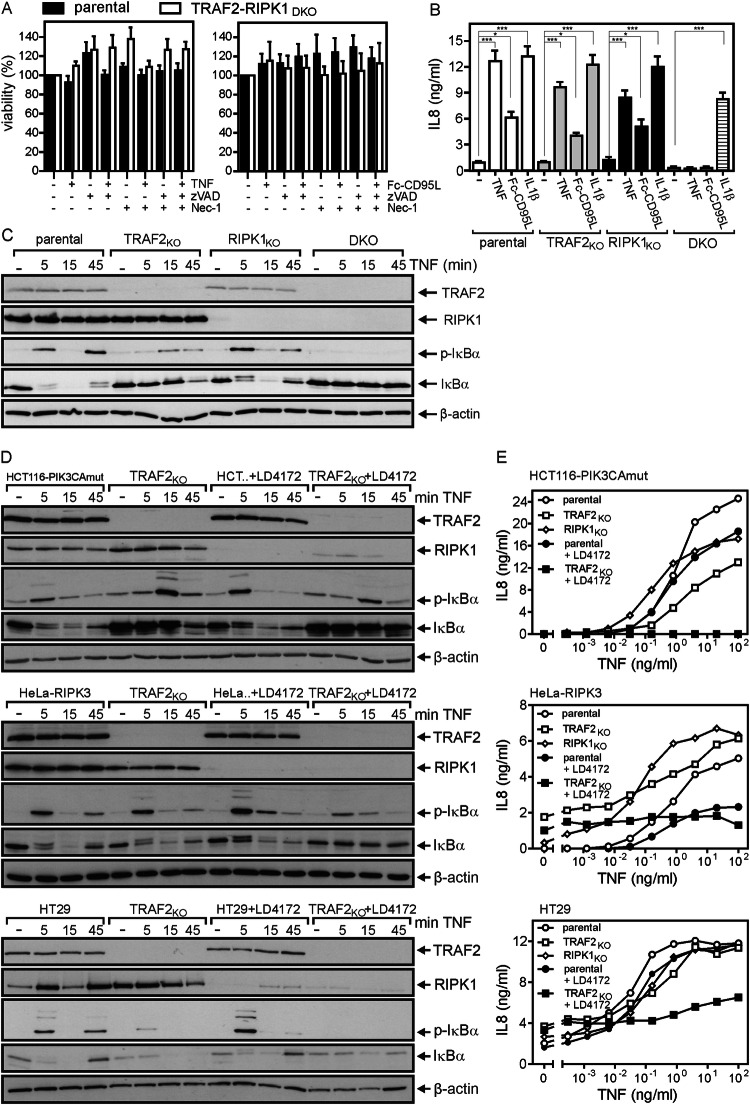
TNF- and CD95L-induced secretion of the classical NFκB target IL-8 is completely abrogated in TRAF2-RIPK1 double deficient cells. **A** Cells were stimulated overnight with 100 ng/ml TNF or 200 ng/ml Fc-CD95L and after 16–18 h, cell viability was evaluated by crystal violet staining. Shown are the mean +/− SEM of 5 independent experiments. Cell death induction was statistically evaluated by two-way ANOVA of parental and TRAF2-RIPK1 double-deficient HCT116-PIK3CAmut cells. There were no significant differences. **B** Cells were stimulated overnight with 100 ng/ml TNF, 200 ng/ml Fc-CD95L or 20 ng/ml IL-1β. After 16–18 h, IL-8 production was quantified by ELISA analysis of cell culture supernatants. Shown are the mean +/− SEM of 4 independent experiments. IL-8 production data sets of parental, TRAF2-deficient, RIPK1-deficient and double-deficient cells were statistically evaluated by one-way ANOVA (Bonferroni post-hoc test). ***p < 0.001; *p < 0.05. **C** Cells were stimulated for the indicated times with 100 ng/ml TNF and total cell lysates were evaluated with respect to the presence/phosphorylation of the indicated proteins by western blotting. **D**, **E** TRAF2-deficient variants of HCT116-PIK3CAmut, HeLa-RIPK3 and HT29 cells along with their parental counterparts were treated with 1 µM LD4172 for 24 h and were then analyzed with respect to RIPK1 expression and phosphorylation and degradation of IκBα by western blotting (**D**) and with respect to IL8 induction by ELISA (**E**). One of two (**D**) or three (**E**) experiments with similar results are shown.

It is commonly assumed that RIPK1 acts downstream of TRAF2 in TNF-induced NFκB signaling as the major substrate for K63-ubiquitination by the TRAF2-associated E3 ligases cIAP1 and cIAP2 [7]. In this respect, we observed that TNF- and Fc-CD95L-induced IL-8 secretion were reduced to a variable extent but remained principally intact in RIPK1-deficient variants of HCT116-PIK3CAmut and HT29 cells (Fig. 4D). In the RIPK1-deficient HeLa-RIPK3 cells, there was even a somewhat enhanced IL-8 response (Fig. 4D). Moreover, TNF-induced phosphorylation and degradation of IκBα were largely similar in the HCT116-PIK3CAmut, HeLa-RIPK3 and HT29 cells and the corresponding RIPK1-deficient variants (Fig. 4E). Taken together, our data indicate that RIPK1 acts in a non-obligatory manner in TNF-induced activation of the classical NFκB pathway, therefore resembling the situation in the TRAF2-deficient cell variants. The fact, that both RIPK1- and TRAF2-deficient variants of the same cell line show substantial residual or even normal induction of IL-8 in response to TNF and CD95L suggests that the TRAF2-cIAP1/2-RIPK1 axis is either of only secondary relevance for DR-induced NFκB signaling or opens the possibility that TRAF2 and RIPK1 act independently from each other in separate DR-induced signaling pathways culminating in classical NFκB signaling. To evaluate the latter idea, we analyzed HCT116-PIK3CAmut and HCT116-PIK3CAmut-TRAF2_KO_ cells deficient for RIPK1 (HCT116-PIK3CAmut-RIPK1_KO_, HCT116-PIK3CAmut-TRAF2/RIPK1_DKO_). In accordance with the fact that DR-induced apoptosis signaling is blocked in HCT116-PIK3mut cells downstream of caspase-8 activation [9, 24], resistance against TNF- and CD95L-induced apoptosis remained high in HCT116-PIK3CAmut-TRAF2/RIPK1_DKO_ cells (Fig. 5A). Similarly to HCT116-PIK3CAmut-TRAF2_KO_ cells, TNF- and CD95L-induced IL-8 production remained robust in HCT116-PIK3CAmut-RIPK1_KO_ cells (Fig. 5B). Most intriguingly, however, TNF- as well as CD95L-induced IL-8 production was completely abrogated in the HCT116-PIK3CAmut-TRAF2/RIPK1_DKO_ cells (Fig. 5B). The lack of IL-8 induction in TNF- and CD95L-treated HCT116-PIK3CAmut-TRAF2/RIPK1_DKO_ cells did not reflected a general defect in classical NFκB signaling as this response was still efficiently triggered by IL-1ß (Fig. 5B). Similar to TRAF2 deficiency, RIPK1 deficiency also attenuated TNF-induced phosphorylation and degradation of IκBα (Fig. 5C). In HCT116-PIK3CAmut-TRAF2/RIPK1_DKO_ cells, however, TNF completely lost its ability to induce phosphorylation and degradation of IκBα (Fig. 5C). To further evaluate the TRAF2-RIPK1 redundancy, we took advantage of the RIPK1-specific proteolysis targeting chimera (PROTAC) LD4172 [25]. Treatment of the cells with this RIPK1 degrading compound for 24 h resulted in efficient reduction of RIPK1 expression in parental and TRAF2-deficient HCT116-PIK3CAmut, HeLa-RIPK3 and HT29 cells (Fig. 5D). More importantly, LD4172 treatment phenocopied the effects of RIPK1-deficiency on parental and TRAF2-deficient HCT116-PIK3CAmut cells with respect to TNF-induced NFκB signaling (Fig. 5D, E). Treatment with LD4172 also phenocopied the effects of RIPK1-deficiency in HeLa-RIPK3 and HT29 cells. Comparing all three cell lines, TNF-induced NFκB signaling remained largely unaffected in the parental LD4172-treated cells but was practically abrogated in the LD4172-treated TRAF2-deficient cell line variants (Fig. 5D, E). Thus, redundant TRAF2- and RIPK1-mediated TNF-induced NFκB signaling appear to be a general phenomenon.

**Fig. 6:**
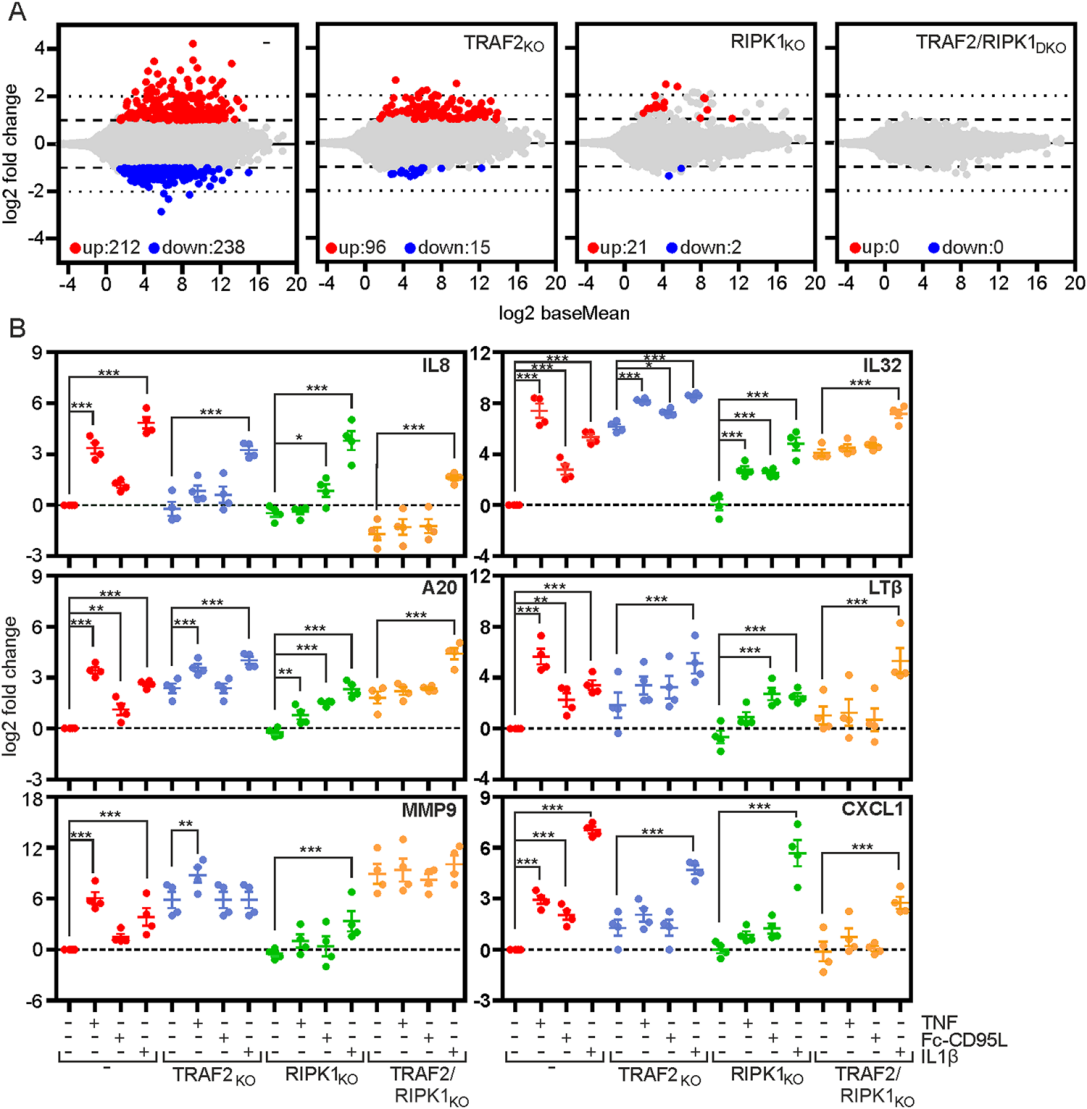
Death receptor-induced, but not IL-1β-induced gene induction is severely affected in TRAF2-RIPK1 double deficient cells. **A** The indicated HCT116-PIK3CAmut cell line variants were challenged with TNF (100 ng/ml, 18 h) and analyzed by NGS. **B** Cells were stimulated overnight (16–18 h) with 100 ng/ml TNF, 200 ng/ml Fc-CD95L or 20 ng/ml IL-1β to prepare total RNA. The latter was reverse transcribed to cDNA and analyzed by qPCR with respect to the expression of the indicated mRNAs (normalized to *RPLP0*). qPCR data shown are derived from 4 independent experiments and were statistically evaluated by one-way ANOVA (Bonferroni post-hoc test). ***p < 0.001; **p < 0.01; *p < 0.05.

To obtain a broader and more unbiased insight into the relevance of TRAF2 and RIPK1 for gene inductive TNF signaling, we analyzed the parental HCT116-PIK3CAmut cell line as well as the TRAF2-, RIPK1- or double-deficient variants with respect to TNF-induced gene induction by next generation sequencing. 212 genes were significantly upregulated (>2-fold) in TNF-treated parental HCT116-PIK3CAmut cells and still 96 or 21 genes upregulated (>2-fold) in the HCT116-PIK3CAmut-TRAF2_KO_ and HCT116-PIK3CAmut-RIPK1_KO_ cells, respectively (Fig. 6A). However, in the TNF-treated TRAF2/RIPK1 double-deficient cells not a single gene was detected with > 2-fold changed expression level, which reflects the quality cut-off used for evaluation of the NGS data (Supplementary Table S1; Fig. 6A). The same pattern applied with target genes down-regulated in response to TNF (Fig. 6A). The overwhelming majority of the top upregulated genes in the TNF-treated HCT116-PIK3CAmut, HCT116-PIK3CAmut-TRAF2_KO_ and HCT116-PIK3CAmut-RIPK1_KO_ cells identified by NGS are known NFκB targets. With exception for SAA1, SAA2 and the naturally occurring read-through transcription of SAA2-SAA4, the inhibitory effect on TNF-induced RNA expression was more severe in RIPK1- than in TRAF2-deficient cells (Table 1). None of these genes was significantly upregulated in HCT116-PIK3CAmut-TRAF2/RIPK1_DKO_ cells (Table 1). Quantitative RT-PCR analysis of selected targets fully confirmed the results obtained by NGS (Fig. 6B). The expression of the targets selected for qPCR was also investigated in response to Fc-CD95L. Although induction was typically lower compared to the TNF response and did not reach significance for every target gene, the overall regulation pattern was the same, partial inhibition in the single KO variants and full abrogation of the response in the DKO cells (Fig. 6B). Again, the IL1ß-induced response remained largely unaffected (Fig. 6B). In sum, these findings clearly show that gene induction by TNFR1 and CD95 in HCT116-PIK3CAmut cells is fully dependent from TRAF2 and RIPK1 but can be mediated alone by each of these molecules, thus in the absence of hierarchical TRAF2-RIPK1 cooperation. It is worth mentioning that the basal expression of many but not all targets analyzed were already significantly upregulated in the TRAF2-deficient and TRAF2/RIPK1-double deficient cell variants but not in the RIKP1-deficient cells (Fig. 6B). For example, there was no major effect of TRAF2 deficiency on basal IL-8 production in HCT116-PIK3CAmut cells neither at the protein nor the mRNA level (Figs. 2A, 5B and 6B), while the constitutive expression of A20, IL32 and several more targets was significantly upregulated (Fig. 6B). It is tempting to speculate that this reflects the activation of the alternative NFκB pathway that is associated with TRAF2-deficiency [12, 13], but this was not further investigated here.

**Table 1:** Top regulated genes in TNF-treated HCT116-PIK3CAmut variants identified by NGS.

Gene ID	Name	Description	log2 fold change (unstimulated vs. 100 ng/ml TNF 18 h)			
			Parental	TRAF2-KO	RIPK1-KO	DKO
9235	IL32	Interleukin 32	4.22	1.76	0.94	n.s.
3604	TNFRSF9	TNF receptor superfamily member 9	3.53	1.87	n.s.	n.s.
4050	LTB	Lymphotoxin beta	3.48	1.62	0.97	n.s.
330	BIRC3	Baculoviral IAP repeat containing 3	3.38	1.78	0.73	n.s.
7185	TRAF1	TNF receptor associated factor 1	3.20	2.51	n.s.	n.s.
8605	PLA2G4C	Phospholipase A2 group IVC	3.11	1.16	1.03	n.s.
6288	SAA1	Serum amyloid A1	2.95	1.04	2.37	n.s.
6352	CCL5	C-C motif chemokine ligand 5	2.72	1.50	1.38	n.s.
3656	IRAK2	Interleukin 1 receptor associated kinase 2	2.71	1.80	0.63	n.s.
5971	RELB	RELB proto-oncogene, NF-kB subunit	2.71	1.25	1.03	n.s.
1437	CSF2	Colony stimulating factor 2	2.68	1.93	0.77	n.s.
3576	CXCL8/IL8	C-X-C motif chemokine ligand 8	2.67	1.40	n.s.	n.s.
6364	CCL20	C-C Motif Chemokine Ligand 20	2.64	2.20	n.s.	n.s.
4792	NFKBIA	NFKB inhibitor alpha	2.62	1.68	0.68	n.s.
6289	SAA2	Serum amyloid A2	2.61	0.87	2.46	n.s.
8710	SERPINB7	Serpin family B member 7	2.51	2.06	0.84	n.s.
7127	TNFAIP2	TNF alpha induced protein 2	2.50	1.20	0.64	n.s.
89795	NAV3	Neuron navigator 3	2.49	1.65	n.s.	n.s.
972	CD74	CD74 molecule	2.40	1.45	0.68	n.s.
629	CFB	Complement factor B	2.39	0.69	0.81	n.s.
22806	IKZF3	IKAROS family zinc finger 3	2.38	1.29	n.s.	n.s.
79155	TNFAIP3	TNFAIP3 interacting protein 2	2.34	1.43	n.s.	n.s.
2919	CXCL1	C-X-C motif chemokine ligand 1	2.20	1.67	n.s.	n.s.
100528017	SAA2-SAA4	SAA2-SAA4 readthrough	2.20	0.79	1.97	n.s.
2920	CXCL2	C-X-C motif chemokine ligand 2	2.18	1.33	n.s.	n.s.
81788	NUAK2	NUAK family kinase 2	2.14	1.39	n.s.	n.s.
10537	UBD	Ubiquitin D	2.12	1.51	0.69	n.s.
10148	EBI3	Epstein-Barr virus induced 3	2.10	1.59	0.78	n.s.
6648	SOD2	Superoxide dismutase 2	2.10	0.58	0.41	n.s.
51561	IL23A	Interleukin 23 subunit alpha	2.10	2.05	n.s.	n.s.
4790	NFKB1	Nuclear factor kappa B subunit 1	2.07	1.19	0.50	n.s.
718	C3	Complement C3	2.06	0.90	0.97	n.s.
54839	LRRC49	Leucine rich repeat containing 49	2.06	1.65	0.56	n.s.
89797	NAV2	Neuron navigator 2	2.04	0.93	n.s.	n.s.
3659	IRF1	Interferon regulatory factor 1	2.01	1.67	0.50	n.s.
4312	MMP1	Matrix metallopeptidase 1	0.85	2.23	n.s.	n.s.
3398	ID2	Inhibitor of DNA binding 2	−2.14	−1.00	n.s.	n.s.
25840	METTL7A	Methyltransferase like 7A	−2.31	−1.14	n.s.	n.s.
2302	FOXJ1	Forkhead box J1	−2.85	−1.23	−1.21	n.s.

n.s. = padj > 0.05, log2 fold change > 2.

### TRAF2 and RIPK1 are needed for complex I formation but are dispensable for DISC formation

**Fig. 7:**
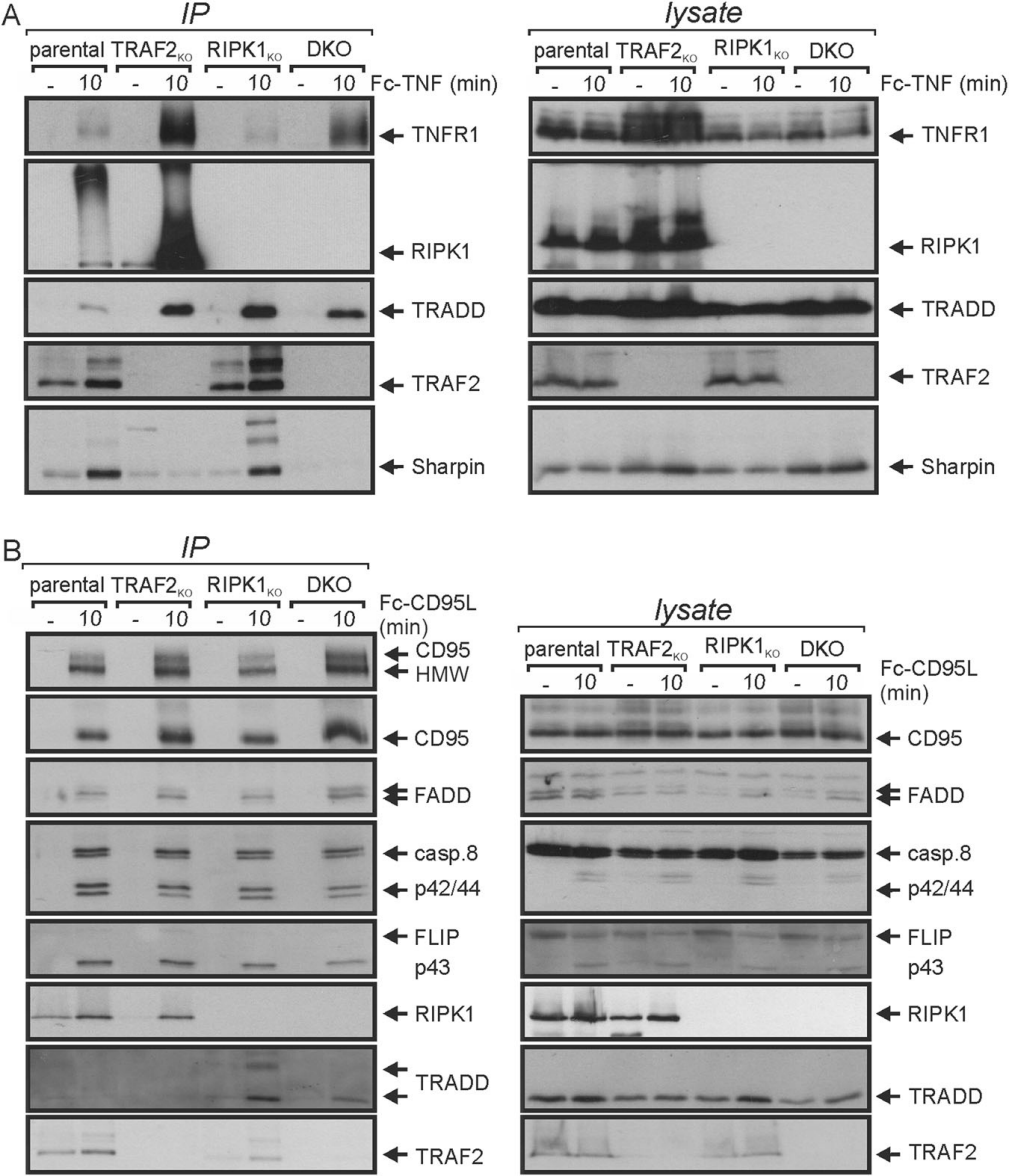
RIPK1 antagonizes CD95L-induced DISC recruitment of the TRADD-TRAF2 dyad. Ligand-induced TNFR1- (**A**) and CD95-associated (**B**) signaling complexes were immunoprecipitated from the indicated HCT116-PIK3CAmut cell line variants via their respective Fc-linked ligands and co-precipitated proteins were analyzed by western blotting.

The TNF-induced IKK-stimulating default signaling complex of TNFR1, often called complex I, contains besides TRADD, TRAF2, RIPK1 and the TRAF2-associated E3 ligases cIAP1 and cIAP2, also the sharpin-containing LUBAC, the TAB2/TAK1 complex and the IKK complex [26]. In accordance with our data showing that neither TRAF2 nor RIPK1 alone are obligate for TNFR1-induced NFκB activation/gene transcription, TNF induced a “partial” complex I in HCT116-PIK3CAmut-TRAF2_KO_ and HCT116-PIK3CAmut-RIPK1_KO_ cells (Fig. 7A). The main differences in the TNFR1 signaling complexes between HCT116-PIK3CAmut and HCT116-PIK3CAmut-TRAF2_KO_ cells, was, aside the trivial absence of TRAF2, the strongly reduced appearance of modified RIPK1 species and diminished recruitment of the LUBAC component sharpin (Fig. 7A). The latter is in good accordance with the fact that TRAF2-associated cIAPs K63-ubiquitinate TNFR1-bound RIPK1 to enable LUBAC recruitment [7, 25, 26]. Interestingly, there was significant recruitment of the LUBAC subunit sharpin to the TNFR1 signaling complex in the RIPK1-deficient cell line variant. It is tempting to speculate that here K63-ubiquitinated TRAF2 [27] replaces K63-ubiquitinated RIPK1 as an anchor for LUBAC recruitment.

Most importantly, however, and in accordance with the functional data shown in Figs. 5 and 6, with exception of the direct TNFR1 binder TRADD, none of the complex I components analyzed were recruited into the TNFR1 signaling complex of HCT116-PIK3CAmut-TRAF2/RIPK1_DKO_ cells (Fig. 7A).

The CD95L-induced default signaling complex of CD95, often called death inducing signaling complex (DISC), contains FADD, caspase-8 and FLIP. In all four HCT116-PIK3CAmut variants, Fc-CD95L efficiently induced formation of this complex without significant changes indicating that neither TRAF2 or RIPK1 nor both molecules in concert play a role in the interaction of CD95 with these factors (Fig. 7B). Worth mentioning, however, recruitment of TRADD and TRAF2 to the CD95 signaling complex, which is hardly detectable in HCT116-PIK3CAmut cells, was regularly observed in the RIPK1-deficient HCT116-PIK3CAmut variant (Fig. 7B). Thus, despite an increased presence of TRADD and TRAF2 in the CD95 signaling complex in the absence of RIPK1 there was no enhanced gene induction (Fig. 5B) emphasizing that both TRAF2 and RIPK1 play a role in this response.

## Discussion

**Fig. 8:**
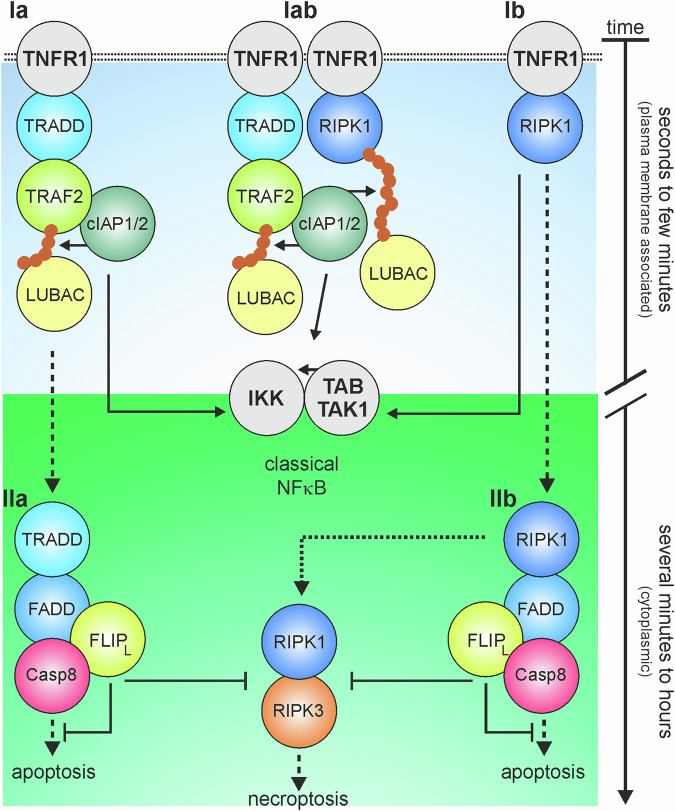
Model of TNF-induced TNFR1-associated signaling complexes in RIPK1- (complex Ia) and TRAF2-deficient (complex Ib) cells and their interplay (complex Iab). For details, please see discussion section.

The current expert view on the role of TRAF2 and RIPK1 in TNFR1-induced activation of the classical NFκB pathway is that both molecules act as a single “unit” in a cooperative-hierarchical manner [2, 7, 28–31]. TRAF2 and its interaction partners cIAP1 and cIAP2 indirectly recruit to the TNFR1 signaling complex downstream of TRADD and RIPK1. However, functionally TRAF2 and the cIAPs act upstream of RIPK1 by catalyzing its K63-ubiquitination enabling the recruitment of downstream effector proteins (e.g. TAK1, LUBAC, IKK complex) and eventually the phosphorylation of IκBα. The current view on TRAF2, cIAP1/2 and RIPK1 acting as an intimate unit in a quasi linear fashion to orchestrate TNFR1 – TRADD/RIPK1 – TRAF2/cIAP1/2 - modified RIPK1 – LUBAC - TAK1/IKK complex I assembly, is at first glance in good accordance with functional and biochemical evidence. Indeed, it has been shown that TRADD and RIPK1 compete for TNFR1 binding and are able to independently recruit TRAF2 [5, 32–34]. In the absence of TRADD and RIPK1, however, there is no TRAF2 recruitment at all and also no classical NFκB activation [5]. Furthermore, RIPK1 ubiquitination, evident from excessive “laddering” of modified RIPK1 species in SDS-PAGE analysis of TNFR1 immunoprecipitations, is strongly blocked in the absence of TRAF2, or after inhibition/depletion of cIAPs. Furthermore, NEMO-deficient cells are unable to transduce classical NFκB signaling. However, there is also striking evidence challenging the simple view that TNFR1 induces classical NFκB signaling by one type of complex I and thereby one defined pathway. Early on, significant TNFR1-induced classical NFκB signaling has been observed in cells of TRAF2-deficient mice [22, 35]. However, it was soon shown that TNFR1-induced IκBα degradation is more severely (but not completely) inhibited in murine embryonic fibroblasts originating from TRAF2-TRAF5 double-deficient mice [22]. The model of a single NFκB signaling-inducing TNFR1-associated complex I with TRAF2-cIAP1/2-mediated K63 RIPK1 ubiquitination as a central element was therefore only modified to the point that TRAF5 can act redundantly with TRAF2 in complex I. However, a simple TRAF2-TRAF5 substitution cannot explain why RIPK1 modification in complex I seems to be completely inhibited in TRAF2 KO cells, while IκBα degradation remains only slightly affected. Instead, this observation suggests that RIPK1, even in its unmodified form, can mediate strong activation of the classic NFκB signaling pathway through TNFR1. A simple TRAF2-TRAF5 redundancy is furthermore difficult to reconcile with the early finding that the transgenic expression of a dominant-negative TRAF2 mutant in lymphocytes inhibits TNF-induced JNK activation but does not inhibit TNF-induced NFκB activation [36]. Recruitment of the TRAF5 molecule into the TNFR1 signaling complex has yet not been described, although the TNFR1 signaling complex has been analyzed in numerous publications in recent years. In the study presented here, ectopic expression of TRAF5 does not enhance TNFR1-induced activation of the classical NFκB pathway in either parental or TRAF2-deficient HCT116-PIK3CAmut cells (Fig. 4B, C). Studies of complex I in RIPK1-deficient cells also challenged the view of the central importance of TRAF2-cIAP1/2 and/or TRAF5-cIAP1/2-mediated K63 ubiquitination of RIPK1 for the classic NFκB-stimulating function of complex I. It was shown early on that in RIPK1-deficient cells TNFR1 still activates the classic NFκB signaling pathway [37], a finding matched by the observation in our present study that classical NFκB signaling is still robustly induced by TNF in three different RIPK1-deficient cell lines (Fig. 4D, E). We therefore suggest a modified model of TNFR1-induced complex I-mediated NFκB signaling which is consistent with the existing experimental evidence and which considers two distinguishable types of complex I in TNF-induced NFκB signaling: First, a TRADD-TRAF2/cIAP utilizing complex Ia which becomes evident in RIPK1-deficient cells. Second, a non-modified RIPK1 utilizing complex Ib which can rescue TRADD- or TRAF2-deficient cells. Complex Ia and Ib may furthermore interact and cooperate to K63 ubiquitinate RIPK1 resulting in a modified complex Iab preventing complex Ia or Ib to promote cell death signaling (Fig. 8). Indeed, the concept that complex I can come in two flavors mirrors the current view of the field with regard to TNFR1-induced apoptosis. Similarly to the here proposed classical NFκB activation, different TNFR1-induced types of cytoplasmic complexes containing TRADD or alternatively RIPK1 can transduce apoptotic TNF signaling (complex IIa and IIb, Fig. 8). The concept of two types of complex I alternatively interacting with TNFR1 does not only fit with the data reported in the literature, but is also in good accordance with our novel finding that TRAF2-RIPK1 double deficient cells show a complete shut-down of TNF-induced classical NFκB signaling (Figs. 5–7). Future studies must now show whether complex Ia, Ib and Iab (equivalently IIa and IIb) exist as structurally distinguishable complexes of defined composition and stoichiometry or whether these complexes rather represent different states of a single type of a less structurally defined “fluid” complex of various non-obligatory components.

It is tempting to speculate that complex Ia, Ib and Iab-related mechanisms of classical NFκB signaling also operate in the signal transduction of other death receptors. Indeed, we found that classical NFκB signaling induced by CD95 is also redundantly mediated by TRAF2 and RIPK1 (Fig. 5B). Moreover, it appeared that both TRADD and TRAF2 are recruited to CD95 more efficiently in RIPK1-deficient than in parental HCT116-PIK3CAmut cells (Fig. 7B). TRADD tightly interacts with its N-terminal part with the TRAF domain of TRAF2 [38]. Thus, for CD95 signaling it appears possible that TRADD and RIPK1 compete for interaction with CD95 and/or FADD in a similar fashion as in TNFR1 signaling. However, it cannot be ruled out that the observed enhanced presence of the TRADD in the CD95 signaling complex is due to its reduced release from the DISC in the absence of RIPK1. In line with the idea that TRADD/TRAF2 act hierarchically in parallel to RIPK1 in DR-induced NFκB signaling, we observed in a former study that IL-8-induction via TNFR1 and the CD95-related TRAIL death receptors is fully inhibited in TRADD-RIPK1 double-deficient cells, but not in variants only expressing one of the two molecules [5].

## Materials and methods

### Cell lines and reagents

The following cell lines and cell line variants have been described elsewhere: HCT116-PIK3CAmut (ref. [9]; kind gift of B. Vogelstein, Johns Hopkins University, Baltimore, USA), HeLa-RIPK3 (ref. [17]; kind gift of M. Leverkus, University Hospital Aachen, Germany), HT29-RIPK1_KO_ (ref. [39]; kind gift of J. Silke, The Walter and Eliza Hall Institute, Melbourne, Australia), HeLa-RIPK3-RIPK1_KO_ [5], HCT116-PIK3CAmut-TRAF2_KO_ [15], SK-MEL-23-TRAF2_KO_ and D10-TRAF2_KO_ [10]. To generate HCT116-PIK3CAmut-RIPK1_KO_ and HCT116-PIK3CAmut-TRAF2/RIPK1_DKO_ cells, HCT116-PIK3CAmut and HCT116-PIK3CAmut-TRAF2_KO_ cells were transfected using PEI [40] with a 1:1 mixture of the RIPK1 CRISPR/Cas9 KO plasmid mix (Santa Cruz, sc-400377) and the corresponding RIPK1 HDR plamid mix (Santa Cruz, sc-400377-HDR) encoding puromycin, red fluorescent protein and template sequences for homology-directed DNA repair (HDR) of the RIPK1 CRISPR/Cas9 KO plasmid induced DNA cuts. After selection with 1 µg/ml puromycin (2-3 week) clones were isolated and controlled for RIPK1 deficiency. Similarly, HeLa-RIPK3-TRAF2_KO_ and HT29-TRAF2_KO_ were generated from HeLa-RIPK3 and HT29 cells using the TRAF2 CRISPR/Cas9 KO plasmid mix (Santa Cruz, sc-400361 and the corresponding TRAF2 HDR plamid mix (Santa Cruz, sc-400361-HDR). Cells were cultured in RPMI 1640 medium supplemented with 10% fetal calf serum (FCS; Gibco) at 37 °C at 5% CO_2_. TNF was a kind gift of Daniela Männel (University of Regensburg), Killer-TRAIL (TRAIL) was purchased from Enzo Life Sciences and IL-1β was from R&D (201-LB). Fc-Flag-TNF (Fc-TNF) and Fc-Flag-CD95L (Fc-CD95L) were produced and purified in house by transient transfection of HEK293 cells using PEI (Polysciences Inc., Warrington, USA) and purification by anti-Flag affinity chromatography as previously described for other Flag-tagged proteins [40]. Development and synthesis of LD4172 has been described elsewhere [25]. Carbobenzoxy-valyl-alanyl-aspartyl-[*O*-methyl]-fluoromethylketone (ZVAD) was from Bachem, necrostatin-1 (Nec-1) and TPCA-1 were from Tocris and MLN4924 and cycloheximide were from Sigma. Antibodies used are anti-ß-actin (Sigma, #A1978-200UL), anti-CD95 (Cell Signaling, #8023), anti-Cyld (Cell Signaling, 8462), anti-caspase-3 (Cell Signaling, #14220), anti-caspase-8 (Enzo Life Science, #ADI-AAM-118E), anti-FADD (Santa Cruz, #sc-271520), anti-FLIP (Adipogene, #AG-20B-0056-C100), anti-IκBα (Cell Signaling, #4814), anti-pIκBα (Cell Signaling, #2859), anti-MLKL (Abcam, #ab194699), anti-pMLKL (Abcam, #ab187091), anti-PARP (BD, #556494), anti-RIPK1 (BD, 610459), anti-pRIPK1 (Cell Signaling, #44590), anti-p100/p52 (Merck Millipore, #05-361), anti-sharpin (Abcam, #125188), anti-TNFR1 (Cell Signaling, #3736), anti-TRADD (Cell Signaling, #3684), anti-TRAF2 (Cell Signaling, #4724), anti-TRAF5 (Cell Signaling, #41658).

### Cell viability

All cell lines and cell line variants used in this study grow adherently and were seeded (20 × 10^3^ cells in 100 µl FCS-supplemented medium) in 96-well tissue culture plates for evaluation of cell death induction. Next day, medium was replaced by FCS-containing medium supplemented with the ligands (TNF, Fc-CD95L and TRAIL (Killer-TRAIL)) and reagents (ZVAD, Nec-1)) of interest. After cultivation for 16–18 h, cell viability was quantified using crystal violet staining of the surviving adherent cells. For normalization, optically densities derived of untreated cells were defined as 100% and optically densities obtained from cells treated with a highly cytotoxic mixture (2.5 µg/ml CHX, 400 ng/ml Fc-CD95L, 400 ng/ml TRAIL, 400 ng/ml TNF, 0.03% sodium azide) ensuring complete cell killing were defined as 0% viability.

### IL-8 ELISA

For evaluation of ligand-induced production of IL8, cells were again seeded (20 × 10^3^ cells in 100 µl FCS-supplemented medium) in 96-well tissue culture plates. The following day, medium was replaced by FCS-containing medium supplemented with the reagents of interest. In experiments where TPCA-1 and MLN4924 were included, medium supplemented with these compounds were added first and few minuets later ligands, ZVAD and Nec-1 were added. After incubation for 16–18 h, cell culture supernatants were harvested, cleared by centrifugation and evaluated for their IL-8 content using an IL-8 enzyme-linked immunosorbent assay (ELISA) kit (BD Biosciences, Heidelberg, Germany) according to the manufacturer’s protocol.

### Western blotting

Cells were cultivated overnight in 6-well tissue culture plates (1 × 10^6^ cells in 200 µl FCS-supplemented medium) and were then stimulates as indicated. Afterwards cells were scraped into the medium using a rubber policeman, pelleted and washed once with PBS. Cells pellets were resuspended in 4× Laemmli sample buffer supplemented with complete protease inhibitor (Roche Applied Science) and phosphatase inhibitor mixtures II (Sigma) at a concentration of approx. 1 × 10^6^ cells/180 μl. To enhance cell disintegration and protein solubilization, samples were sonicated (25 s, maximum amplitude, UP100H Ultrasonic Processor, Hielscher, Germany) and heated at 95 °C for 5 min. After clearance by centrifugation, lysates were subjected to separation by SDS-PAGE. After transfer of proteins to nitrocellulose and blocking of free binding sites with 5% w/v dry milk for 1 h, membranes were incubated with the primary antibody of interest. Nitrocellulose membrane-associated antibodies were finally visualized with horseradish peroxidase-conjugated anti-mouse IgG (Dako) or anti-rabbit IgG (Cell Signaling) and the ECL Western blotting detection reagents and analysis system (Amersham Biosciences).

### Immunoprecipitation

The receptor signaling complexes of TNFR1 and CD95 were immunoprecipitated by help of Fc-TNF and Fc-CD95L and protein G beads (Roche). Cells were seeded in 15 cm tissue culture dishes, grown near confluency and then stimulated with 1 µg/ml of Fc-TNF for 10 min or 1 µg/ml of Fc-CD95L for 90 min. Untreated cells served as negative control. After stimulation cells were washed three times with ice-cold PBS to remove unbound Fc-TNF/Fc-CD95L. Cells were harvested using a rubber policeman and 10 ml ice-cold PBS. After collecting the cells by centrifugation, the cell pellet were resuspended in 1.5 ml lysis buffer (30 mM Tris HCl pH 7.5, 120 mM NaCl, 1% Triton X-100, 10% glycerol, cOmplete^TM^ protease inhibitor protease cocktail (Sigma-Aldrich) one tablet per 25 ml) and incubated on ice for 20 min. Insoluble debris were removed by two rounds of centrifugation (14,000 × *g*, 20 min). Fc-TNF (5 ng/ml) and Fc-CD95L (10 ng/ml) are added to the lysates obtained from the untreated control cells. All lysates were then supplemented with 40 µl protein G agarose. After overnight incubation at 4 °C with gentle agitation, the protein G beads were collected by centrifugation for 30 s at 500 × *g* and washed four times in lysis buffer. After the last wash, the protein G agarose beads were resuspended in 140 µl Lämmli sample buffer and heated for 15 min at 75 °C. Protein G agarose was again pelleted by centrifugation and the cleared supernatants were subjected to western blotting.

### RNA isolation, qPCR and NGS

Cells were seeded in 6-well tissue culture plates (1 × 10^6^ cells in 2 ml FCS-supplemented medium) and were stimulated the next day with the reagents of interest (NGS technical triplicates; qPCR, independent experiments without technical replicates). To prepare total RNA the RNeasy micro kit (Qiagen) according to the protocol of the manufacturer was used. Total RNA samples were used to generate complementary DNA either with the Omniscript RT kit (Qiagen) or the High-Capacity cDNA Reverse Transcription Kit (Thermo Fisher Scientific). Quantitative PCR (qPCR) was performed with SYBR Select Master Mix on a ViiA7 Real-Time PCR System (Thermo Fisher Scientific). Gene expression was calculated by the comparative ΔΔCt-method and normalized to the housekeeping gene *Rplp0*. For all primers, forward and reverse primers were located either across exon-exon boarders or in different exons (Table 2). Melting temperatures were between 58 and 60 °C (Primer Express 3.0, Life Technologies).

**Table 2:** Primer information qPCR.

Gene name Gene reference	Sequence forward primer	Location	Sequence reverse primer	Location
*RPLP0* ENST00000392514.9	CATCTACAACCCTGAAGTGCTTGAT	exon 6	CAATCTGCAGACAGACACTGGC	exon 7
*CXCL8* (=IL-8) ENST00000307407.8	GTGGACCACACTGCGCC	exon 2	CAGTTTTCCTTGGGGTCCAG	exon 3
*IL32* ENST00000528163.6	AGTGGCGGCTTATTATGAGGA	exon 7	TCGGCACCGTAATCCATCTC	exon 8
*TNFAIP3* (=A20) ENST00000612899.5	GGGGCCCGGAGAGGTAA	exon 1	GGAAGGACTTGTTCAGCCATTG	exon 2
*LTB* ENST00000429299.3	CACTTCTCTGGTGACCTTGTTGC	exon 1	CGGCCGTCTCCGTTACC	exon 1/2
*MMP9* ENST00000372330.3	TGAGAACCAATCTCACCGACA	exon 1	TTCGACTCTCCACGCATCTC	exon 2
*CXCL1* ENST00000395761.4	CCGAAGTCATAGCCACACTCAA	exon 2/3	TTCAGGAACAGCCACCAGTG	exon 4

### Statistical analysis

Data normality was tested using Shapiro–Wilk test and the homogeneity of variances assumption for two or more groups were validated using the Levene’s test without exclusion of outliners. When both was checked positively, the GraphPad Prism5 software was used to analyze two or more dependent means with parametric tests (t test, one-way ANOVA, two-way ANOVA, post-hoc tests).

## Supplementary information

supplemental data figures 1 and 2

supplemental data original WBs

Supplemental Table 1

## Data Availability

The original NGS data set is available from Supplementary Table S1. The original full size western blots are shown in the Original Data WB File.
