# Supplementary material for: TRAF2 and RIPK1 redundantly mediate classical NFκB signaling by TNFR1 and CD95-type death receptors
Source: Cell Death Dis. 2025 Jan 21;16(1):35. doi: 10.1038/s41419-024-07325-x (PMC11751453; doi:10.1038/s41419-024-07325-x)
Supplement: Supplementary file 1 — supplemental data figures 1 and 2 [file 41419_2024_7325_MOESM1_ESM.pdf]

SUPPLEMENTAL DATA

TRAF2 and RIPK1 redundantly mediate classical NFκB signaling by TNFR1 and CD95-type death receptors

Jennifer Wagner[1], David Vredevoogd[2], Xin Yu[4], Dong Lu[4], Daniel S. Peeper[2], Heike M. Hermanns[3], Jin Wang[4,5,6], Harald Wajant[1] and Daniela Siegmund[1]

[1] Division of Molecular Internal Medicine, Department of Internal Medicine II, University Hospital Würzburg, Auvera Haus Grombühlstraße 12, 97080, Würzburg, Germany.

[2] Division of Molecular Oncology and Immunology, Oncode Institute, The Netherlands Cancer Institute, Plesmanlaan 121, 1066 CX Amsterdam, the Netherlands.

[3] Division of Hepatology, Department of Internal Medicine II, University Hospital Würzburg, Auvera Haus Grombühlstraße 12, 97080, Würzburg, Germany.

[4] The Verna and Marrs McLean Department of Biochemistry and Molecular Pharmacology, Baylor College of Medicine, Houston, Texas 77030, USA.

[5] Department of Molecular and Cellular Biology, Baylor College of Medicine, Houston, Texas 77030, USA.

[6] Center for NextGen Therapeutics, Baylor College of Medicine, Houston, Texas 77030, USA.

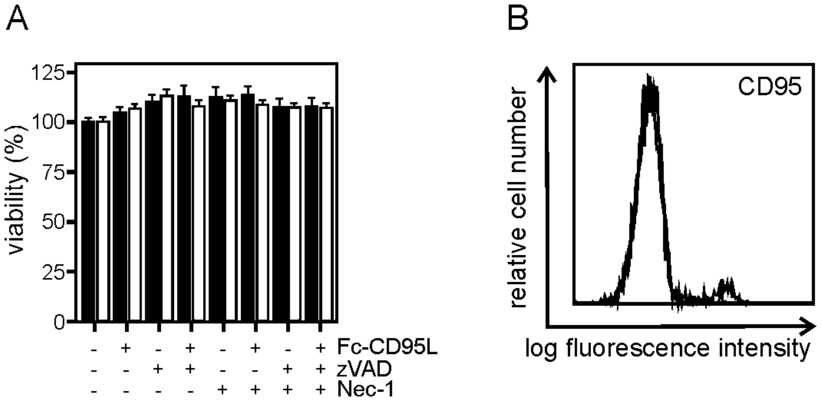

Figure S1. D10 cells do not respond to Fc-CD95L and does not express CD95 on the cell surface.

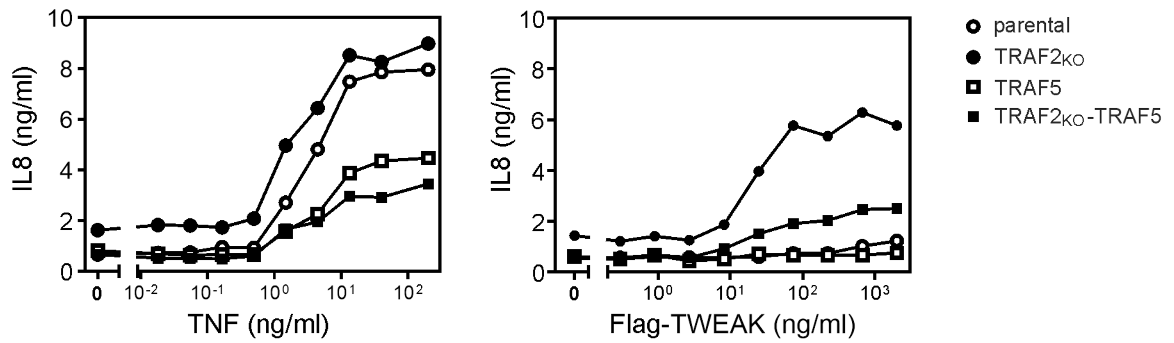

Figure S2. TWEAK-induced IL8 production is enhanced in TRAF5 transfected HCT116-PIK3CAmut cell variants. The indicated HCT116-PIK3CAmut variants were stimulated with increasing concentrations of TNF and Flag-TWEAK. The stimulation with Flag-TWEAK was performed in the presence of 0.5 µg/ml anti-Flag antibody M2 which enhances TWEAK's ability to trigger classical NFκB signaling. After 16-18 hours, IL-8 production was quantified by ELISA analysis of cell culture supernatants. Shown is one representative experiment of three.
