# Supplementary material for: TRAF2 and RIPK1 redundantly mediate classical NFκB signaling by TNFR1 and CD95-type death receptors
Source: Cell Death Dis. 2025 Jan 21;16(1):35. doi: 10.1038/s41419-024-07325-x (PMC11751453; doi:10.1038/s41419-024-07325-x)

ORIGINAL DATA FILE

TRAF2 and RIPK1 redundantly mediate classical NFκB signaling by TNFR1 and CD95-type death receptors

Jennifer Wagner[1], David Vredevoogd[2], Xin Yu[4], Dong Lu[4], Daniel S. Peeper[2], Heike M. Hermanns[3], Jin Wang[4,5,6], Harald Wajant[1] and Daniela Siegmund[1]

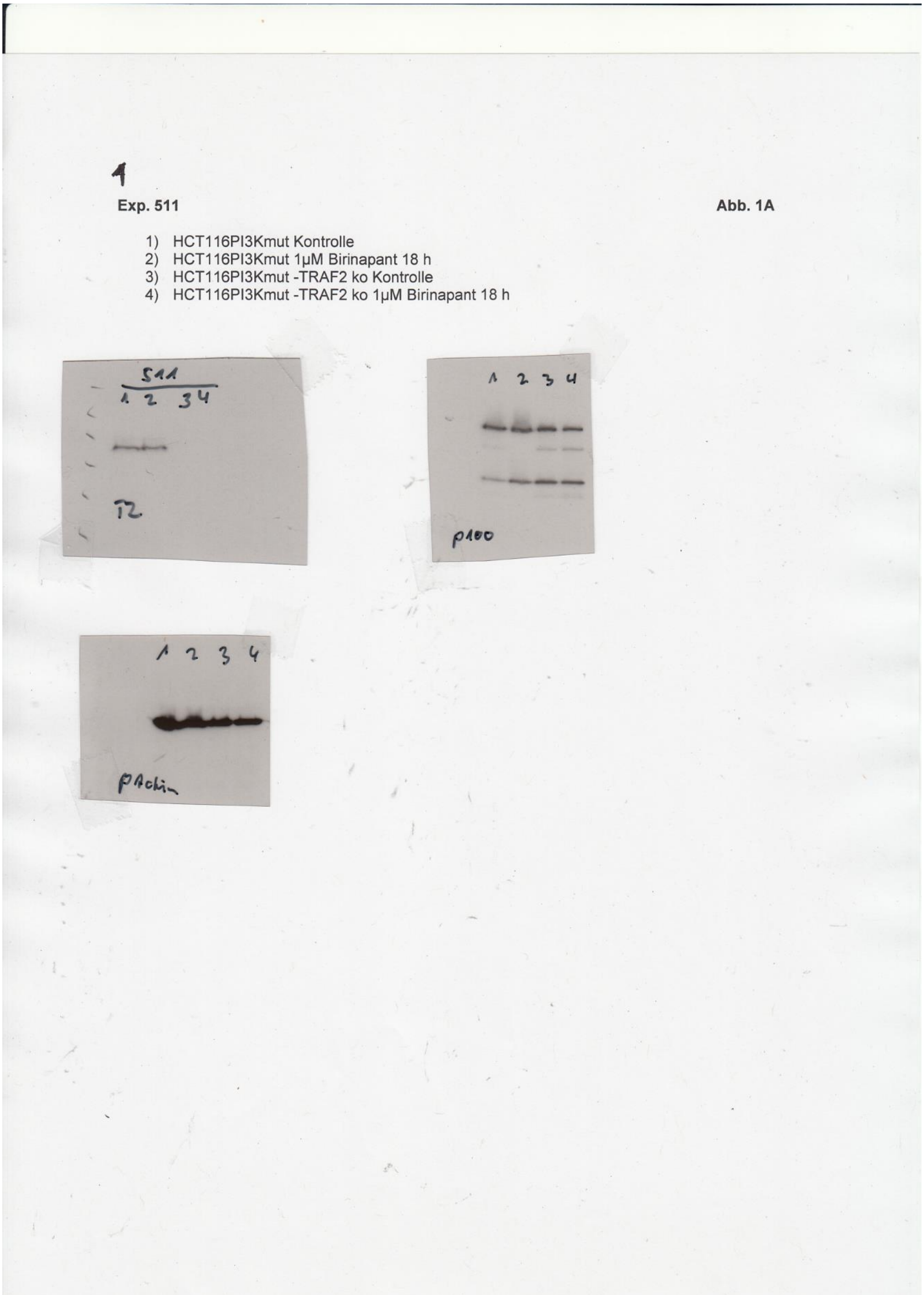

2

Exp. 528 **Abb. 1A**

1) SK-Mel Kontrolle
2) SK-Mel 1µM Birinapant 18 h
3) SK-Mel -TRAF2 ko Kontrolle
4) SK-Mel -TRAF2 ko 1µM Birinapant 18 h

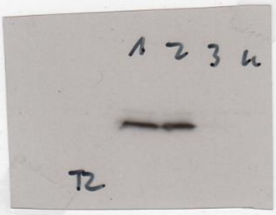

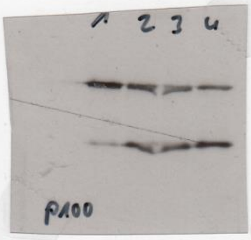

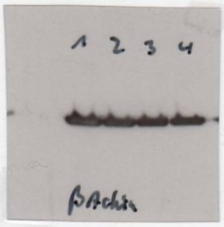

Exp. 509 **Abb. 1A**

1) HeLaRIP3 Kontrolle
2) HeLaRIP3 1µM Birinapant 18 h
3) HeLaRIP3-TRAF2 ko Kontrolle
4) HeLaRIP3-TRAF2 ko 1µM Birinapant 18 h

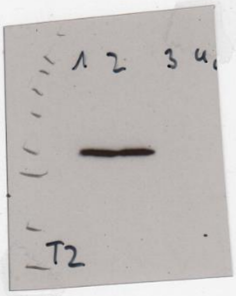

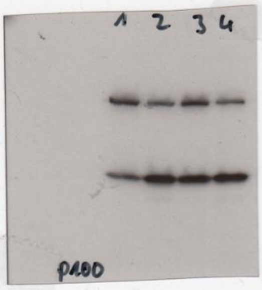

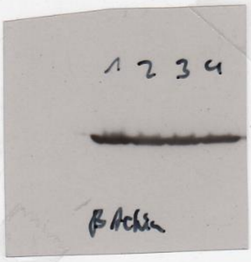

Exp. 510 **Abb: 1A**

1) HT29 Kontrolle
2) HT29 1µM Birinapant 18 h
3) HT29-TRAF2 ko Kontrolle
4) HT29-TRAF2 ko 1µM Birinapant 18 h

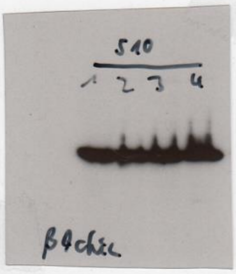

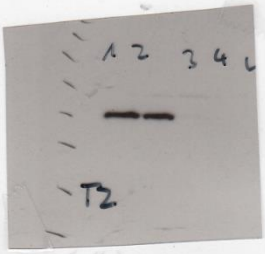

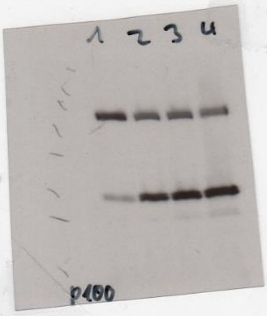

5

Exp. 517 **Abb. 1A**

1) D10 Kontrolle
2) D10 1µM Birinapant 18 h
3) D10-TRAF2 ko Kontrolle
4) D10-TRAF2 ko 1µM Birinapant 18 h

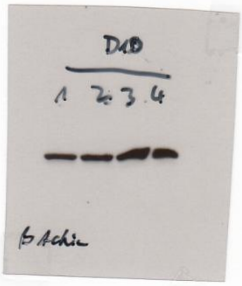

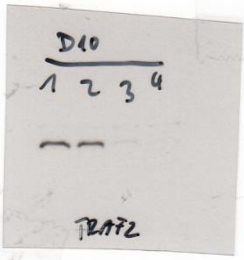

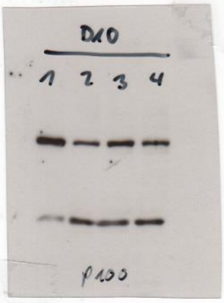

6

Exp. 375

~~Abb. 1B~~
Abb 1C

1) HeLaRIP3 +ZVAD
2) HeLaRIP3+ Z+FasL-Fc 6 h
3) HeLaRIP3 + Z+FasL-Fc 12 h
4) HeLaRIP3+ Z+FasL-Fc 18 h
5) HeLaRIP3-TRAF2 k.o +ZVAD
6) HeLaRIP3-TRAF2 k.o + Z+FasL-Fc 6 h
7) HeLaRIP3-TRAF2 k.o + Z+FasL-Fc 12 h
8) HeLaRIP3-TRAF2 k.o + Z+FasL-Fc 18 h

9) HeLaRIP3 +Necro
10) HeLaRIP3+ N+FasL-Fc 6 h
11) HeLaRIP3 + N+FasL-Fc 12 h
12) HeLaRIP3+ N+FasL-Fc 18 h
13) HeLaRIP3-TRAF2 k.o +Necro
14) HeLaRIP3-TRAF2 k.o + N+FasL-Fc 6 h
15) HeLaRIP3-TRAF2 k.o + N+FasL-Fc 12 h
16) HeLaRIP3-TRAF2 k.o + N+FasL-Fc 18 h

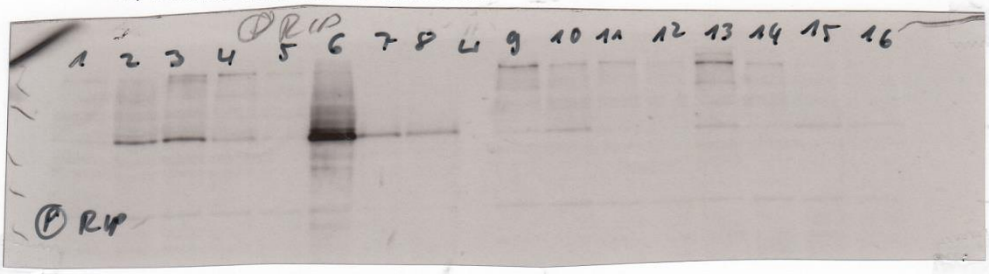

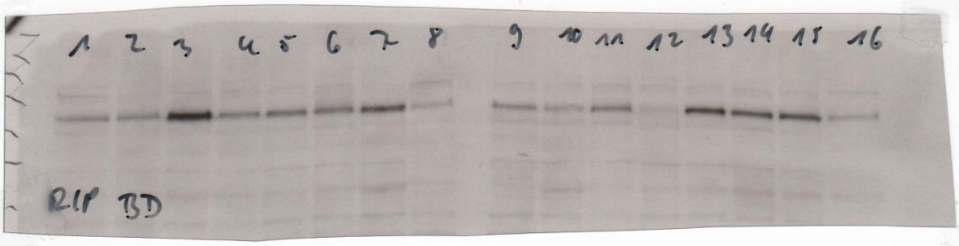

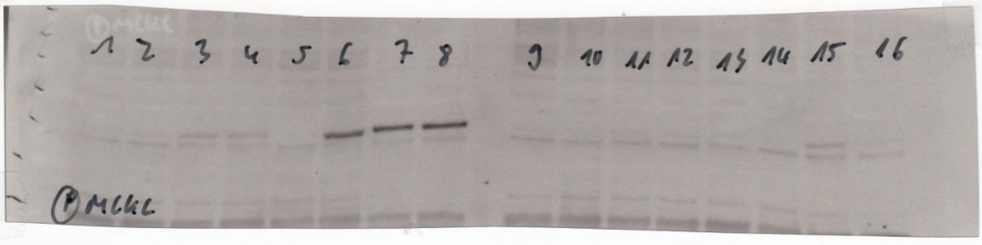

7

Exp. 375

~~Abb. 1B~~

Abb 1C ~~revised~~

1) HeLaRIP3 +ZVAD
2) HeLaRIP3+ Z+FasL-Fc 6 h
3) HeLaRIP3 + Z+FasL-Fc 12 h
4) HeLaRIP3+ Z+FasL-Fc 18 h
5) HeLaRIP3-TRAF2 k.o +ZVAD
6) HeLaRIP3-TRAF2 k.o + Z+FasL-Fc 6 h
7) HeLaRIP3-TRAF2 k.o + Z+FasL-Fc 12 h
8) HeLaRIP3-TRAF2 k.o + Z+FasL-Fc 18 h

9) HeLaRIP3 +Necro
10) HeLaRIP3+ N+FasL-Fc 6 h
11) HeLaRIP3 + N+FasL-Fc 12 h
12) HeLaRIP3+ N+FasL-Fc 18 h
13) HeLaRIP3-TRAF2 k.o +Necro
14) HeLaRIP3-TRAF2 k.o + N+FasL-Fc 6 h
15) HeLaRIP3-TRAF2 k.o + N+FasL-Fc 12 h
16) HeLaRIP3-TRAF2 k.o + N+FasL-Fc 18 h

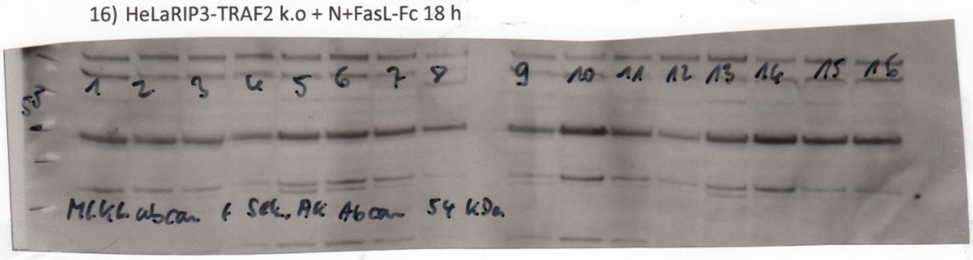

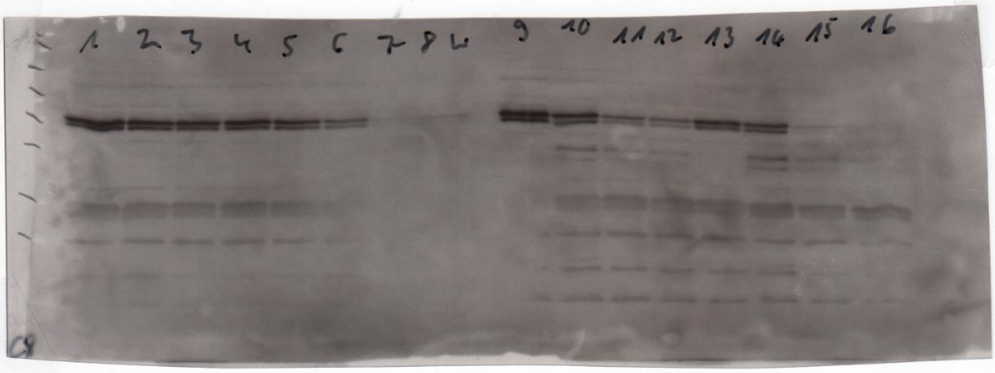

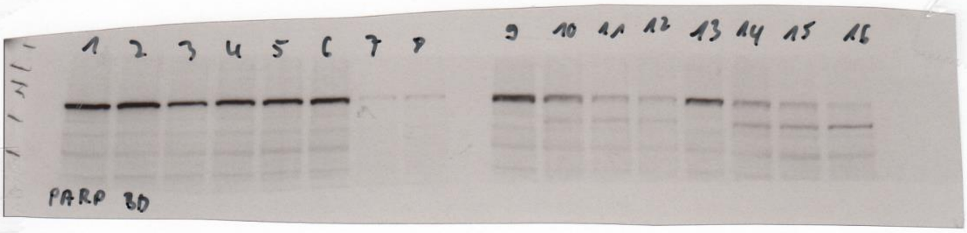

8

Exp. 375

~~Abb. 1B~~
Abb 1C

1) HeLaRIP3 +ZVAD
2) HeLaRIP3+ Z+FasL-Fc 6 h
3) HeLaRIP3 + Z+FasL-Fc 12 h
4) HeLaRIP3+ Z+FasL-Fc 18 h
5) HeLaRIP3-TRAF2 k.o +ZVAD
6) HeLaRIP3-TRAF2 k.o + Z+FasL-Fc 6 h
7) HeLaRIP3-TRAF2 k.o + Z+FasL-Fc 12 h
8) HeLaRIP3-TRAF2 k.o + Z+FasL-Fc 18 h

9) HeLaRIP3 +Necro
10) HeLaRIP3+ N+FasL-Fc 6 h
11) HeLaRIP3 + N+FasL-Fc 12 h
12) HeLaRIP3+ N+FasL-Fc 18 h
13) HeLaRIP3-TRAF2 k.o +Necro
14) HeLaRIP3-TRAF2 k.o + N+FasL-Fc 6 h
15) HeLaRIP3-TRAF2 k.o + N+FasL-Fc 12 h
16) HeLaRIP3-TRAF2 k.o + N+FasL-Fc 18 h

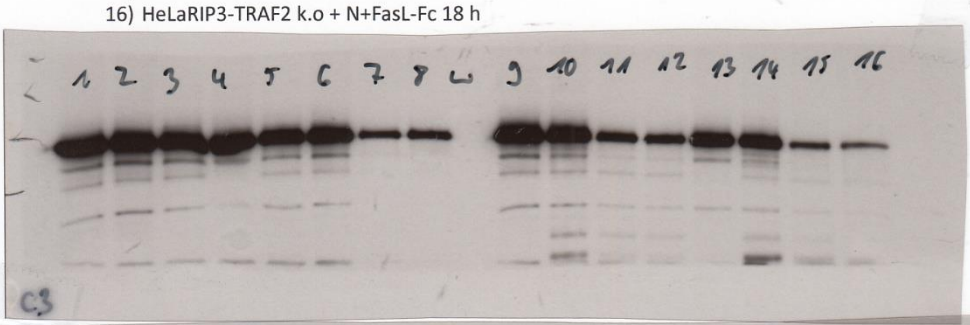

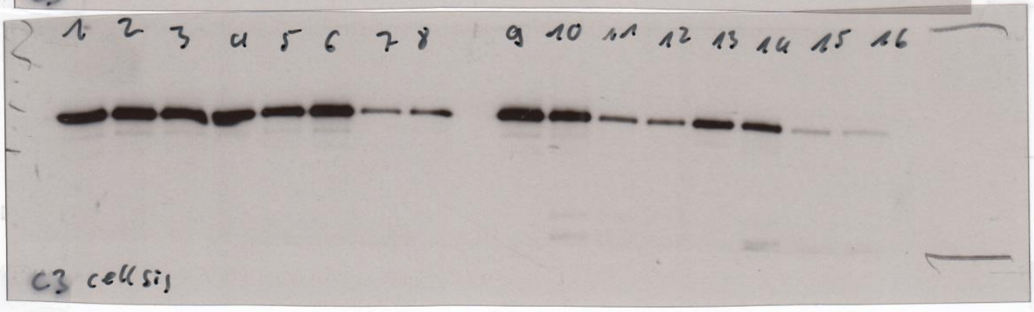

Exp. 375

~~Abb. 1B~~
Abb 1C

1) HeLaRIP3 +ZVAD
2) HeLaRIP3+ Z+FasL-Fc 6 h
3) HeLaRIP3 + Z+FasL-Fc 12 h
4) HeLaRIP3+ Z+FasL-Fc 18 h
5) HeLaRIP3-TRAF2 k.o +ZVAD
6) HeLaRIP3-TRAF2 k.o + Z+FasL-Fc 6 h
7) HeLaRIP3-TRAF2 k.o + Z+FasL-Fc 12 h
8) HeLaRIP3-TRAF2 k.o + Z+FasL-Fc 18 h

9) HeLaRIP3 +Necro
10) HeLaRIP3+ N+FasL-Fc 6 h
11) HeLaRIP3 + N+FasL-Fc 12 h
12) HeLaRIP3+ N+FasL-Fc 18 h
13) HeLaRIP3-TRAF2 k.o +Necro
14) HeLaRIP3-TRAF2 k.o + N+FasL-Fc 6 h
15) HeLaRIP3-TRAF2 k.o + N+FasL-Fc 12 h
16) HeLaRIP3-TRAF2 k.o + N+FasL-Fc 18 h

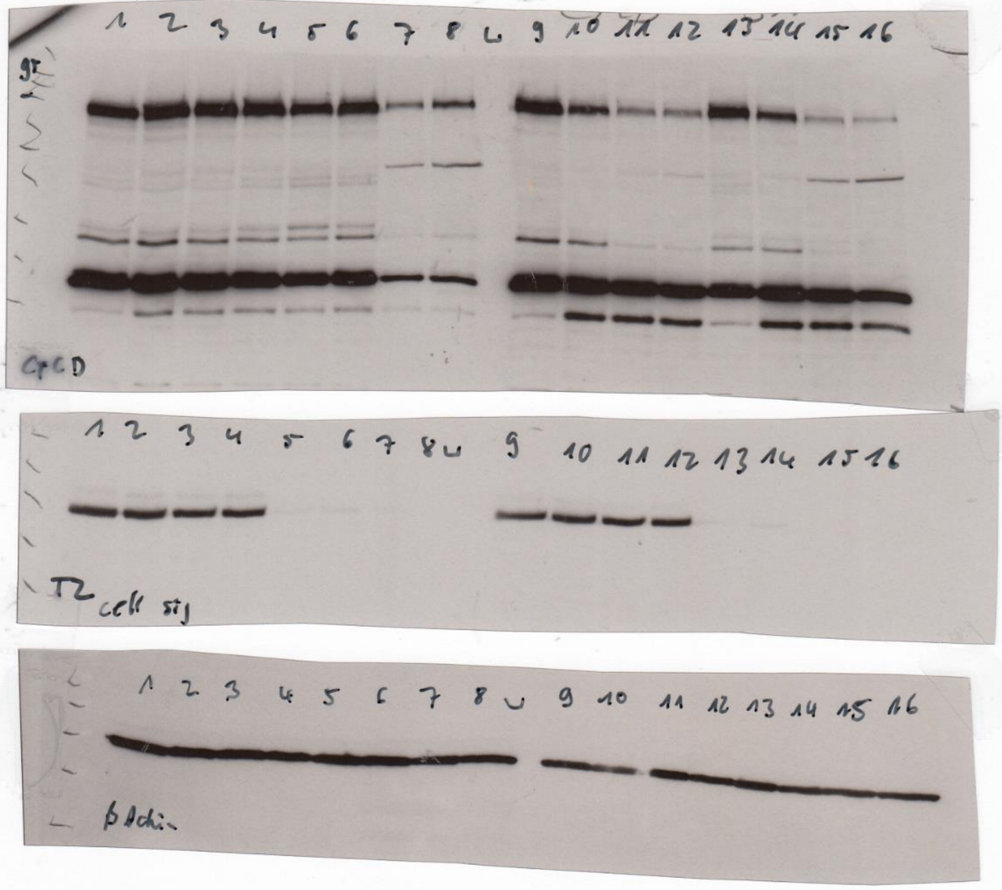

Exp. 374 **Abb. 3**

1) HCT116PI3Kmut Kontrolle
2) HCT116PI3Kmut +TNF 5 min
3) HCT116PI3Kmut + TNF 15 min
4) HCT116PI3Kmut +TNF 1 45 min
5) HCT116PI3Kmut -TRAF2 k.o 10-6 Kontrolle
6) HCT116PI3Kmut -TRAF2 k.o 10-6 TNF + 5 min
7) HCT116PI3Kmut -TRAF2 k.o 10-6 TNF + 15 min
8) HCT116PI3Kmut -TRAF2 k.o 10-6 TNF +45min

9) HCT116PI3Kmut Kontrolle
10) HCT116PI3Kmut +FasL-Fc 1 h
11) HCT116PI3Kmut +FasL-Fc 1 h
12) HCT116PI3Kmut +FasL-Fc 1 h
13) HCT116PI3Kmut -TRAF2 k.o 10-6 Kontrolle
14) HCT116PI3Kmut -TRAF2 k.o 10-6 +FasL-Fc 1 h
15) HCT116PI3Kmut -TRAF2 k.o 10-6 +FasL-Fc 3 h
16) HCT116PI3Kmut -TRAF2 k.o 10-6+FasL-Fc 6 h

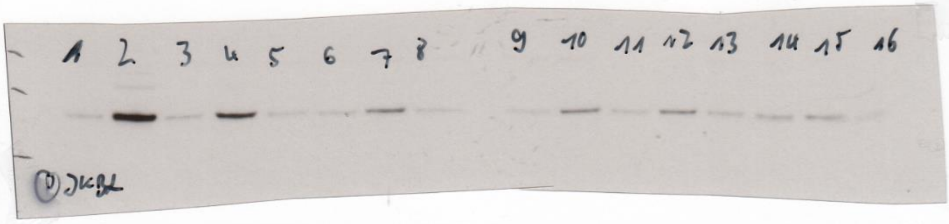

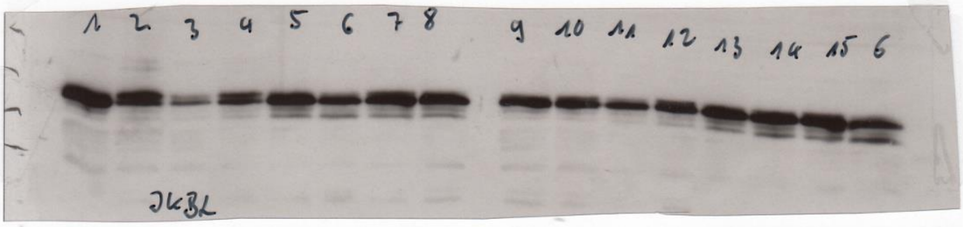

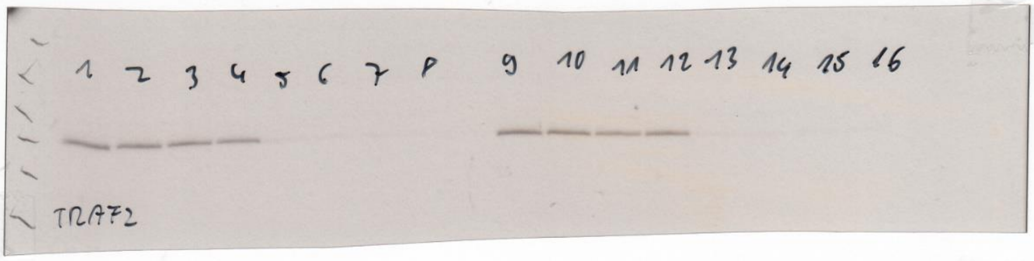

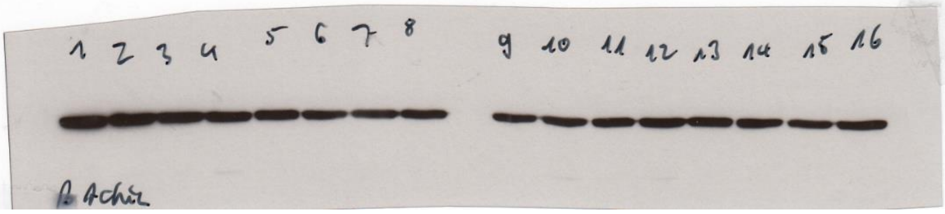

Exp. 372 **Abb. 3**

1) HeLaRIP3 Kontrolle
2) HeLaRIP3+ TNF 5 min
3) HeLaRIP3 +TNF 15 min
4) HeLaRIP3+ TNF 45 min
5) HeLaRIP3-TRAF2 k.o Kontrolle
6) HeLaRIP3-TRAF2 k.o +TNF 5 min
7) HeLaRIP3-TRAF2 k.o +TNF 15 min
8) HeLaRIP3-TRAF2 k.o +TNF 45min

9) HeLaRIP3 Kontrolle
10) HeLaRIP3 +FasL-Fc 1h
11) HeLaRIP3+ FasL-Fc 3h
12) HeLaRIP3 +FasL-Fc 6h
13) HeLaRIP3-TRAF2 k.o Kontrolle
14) HeLaRIP3-TRAF2 k.o +FasL-Fc 1h
15) HeLaRIP3-TRAF2 k.o +FasL-Fc 3h
16) HeLaRIP3-TRAF2 k.o +FasL-Fc 6h

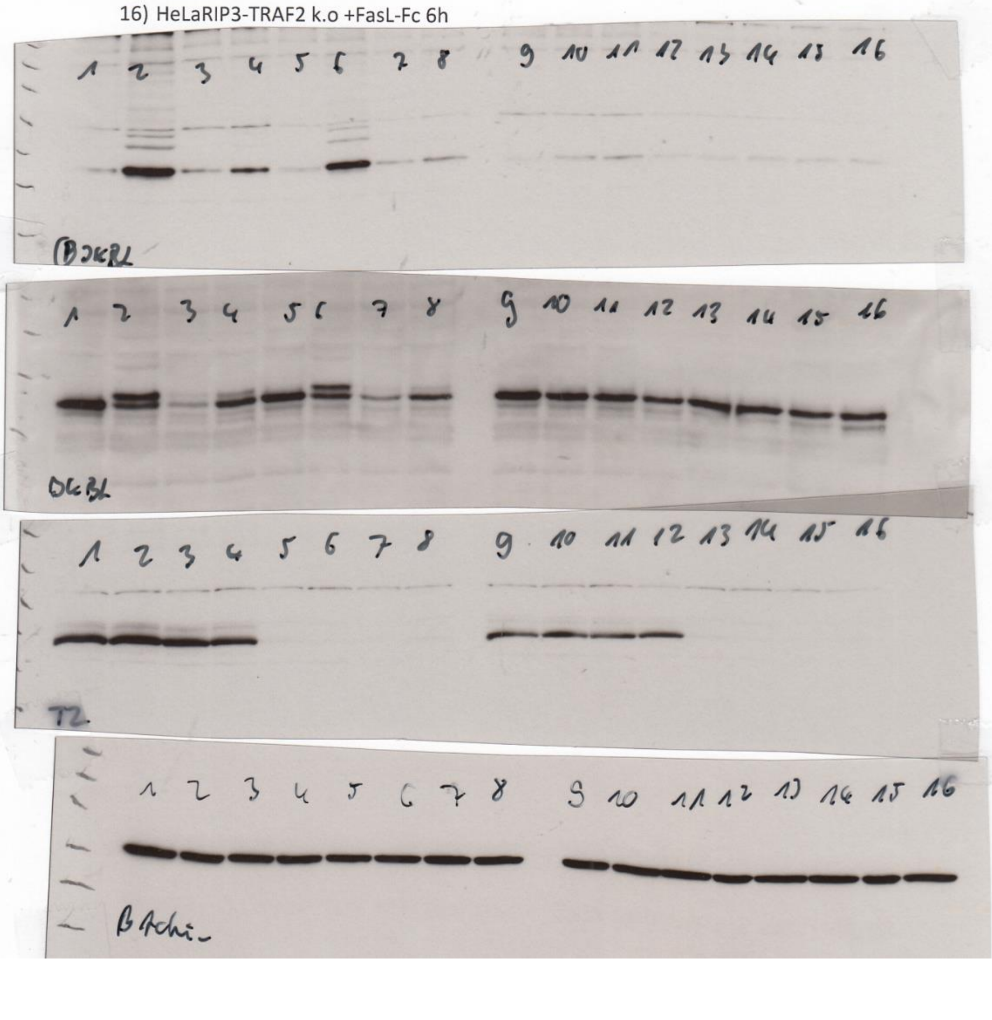

[illegible]

Exp. 373 **Abb.3**

1) HT29 Kontrolle
2) HT29 +TNF 5 min
3) HT29+ TNF 15 min
4) HT29 +TNF 1 45 min
5) HT29-+TRAF2 k.o 10-6 Kontrolle
6) HT29-TRAF2 k.o 10-6 TNF + 5 min
7) HT29-TRAF2 k.o 10-6 TNF + 15 min
8) HT29-TRAF2 k.o 10-6 TNF +45min

9) HT29 Kontrolle
10) HT29 +FasL-Fc 1 h
11) HT29+FasL-Fc 1 h
12) HT29 +FasL-Fc 1 h
13) HT29-TRAF2 k.o 10-6 Kontrolle
14) HT29-TRAF2 k.o 10-6 +FasL-Fc 1 h
15) HT29-TRAF2 k.o 10-6 +FasL-Fc 3 h
16) HT29-TRAF2 k.o 10-6+FasL-Fc 6 h

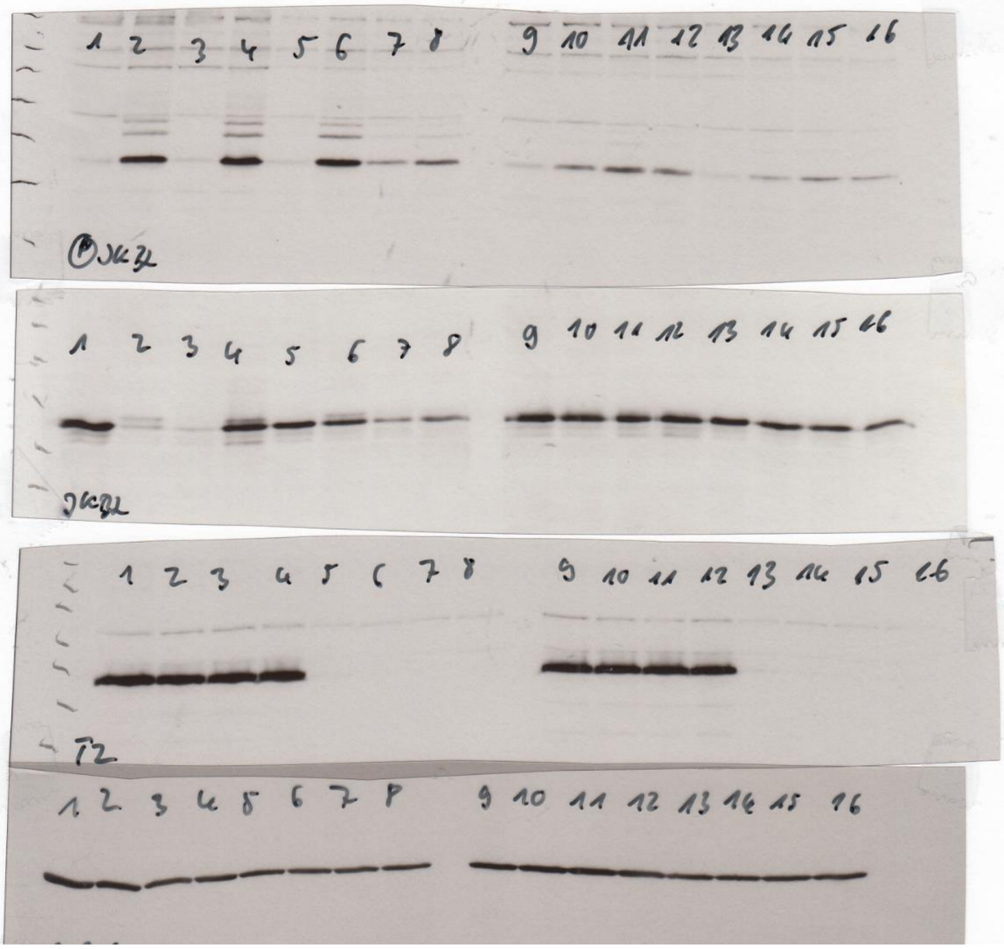

Abb. 4
13

J. Wenzel

Abb 4 A

Nachweis von TRAF2-KO, TRAF1- und TRAF5-Überexpression für Fig. 2

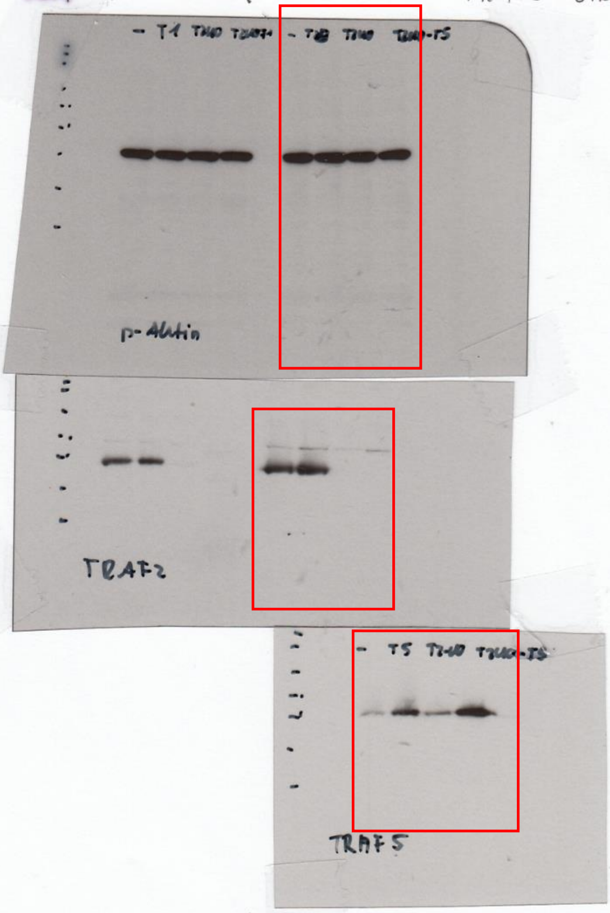

Exp. 532 **Abb. 4C**

1) HCT116PI3Kmut Kontrolle
2) HCT116PI3K mut TNF 5 min
3) HCT116PI3K mut TNF 15 min
4) HCT116PI3K mut TNF 1 45 min
5) HCT116PI3K mut -TRAF2 k.o Kontrolle
6) HCT116PI3K mut -TRAF2 k.o TNF 5 min
7) HCT116PI3K mut -TRAF2 k.o TNF 15 min
8) HCT116PI3K mut -TRAF2 k.o TNF 45min
9) HCT116PI3K mut -TRAF2 ko+TRAF5 Kontrolle
10) HCT116PI3K mut-TRAF2 ko+TRAF5 TNF 5 min
11) HCT116PI3K mut-TRAF2 ko+TRAF5 TNF 15 min
12) HCT116PI3K mut-TRAF2 ko+TRAF5 TNF 45 min
13) HCT116PI3K mut –TRAF5 Kontrolle
14) HCT116PI3K mut –TRAF5 +TNF 5 min
15) HCT116PI3K mut –TRAF5 +TNF 15 min
16) HCT116PI3K mut –TRAF5 +TNF 45min

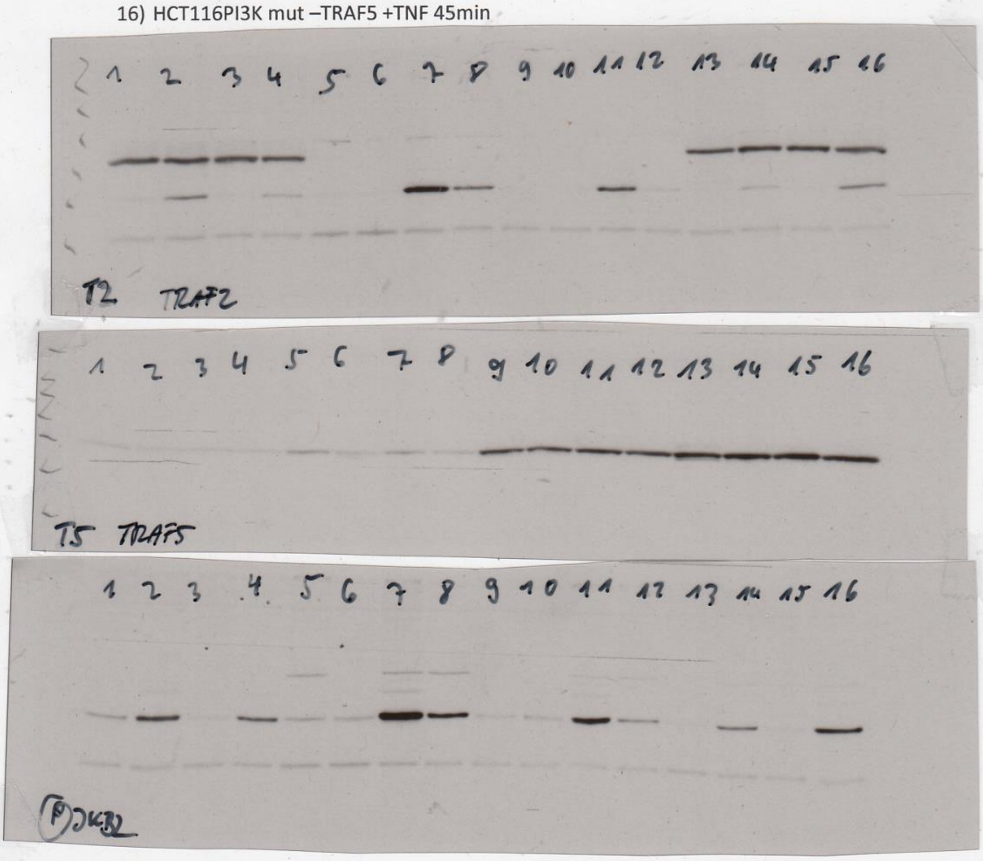

Exp. 532 **Abb. 4C**

1) HCT116PI3Kmut Kontrolle
2) HCT116PI3K mut TNF 5 min
3) HCT116PI3K mut TNF 15 min
4) HCT116PI3K mut TNF 1 45 min
5) HCT116PI3K mut -TRAF2 k.o Kontrolle
6) HCT116PI3K mut -TRAF2 k.o TNF 5 min
7) HCT116PI3K mut -TRAF2 k.o TNF 15 min
8) HCT116PI3K mut -TRAF2 k.o TNF 45min
9) HCT116PI3K mut -TRAF2 ko+TRAF5 Kontrolle
10) HCT116PI3K mut-TRAF2 ko+TRAF5 TNF 5 min
11) HCT116PI3K mut-TRAF2 ko+TRAF5 TNF 15 min
12) HCT116PI3K mut-TRAF2 ko+TRAF5 TNF 45 min
13) HCT116PI3K mut –TRAF5 Kontrolle
14) HCT116PI3K mut –TRAF5 +TNF 5 min
15) HCT116PI3K mut –TRAF5 +TNF 15 min
16) HCT116PI3K mut –TRAF5 +TNF 45min

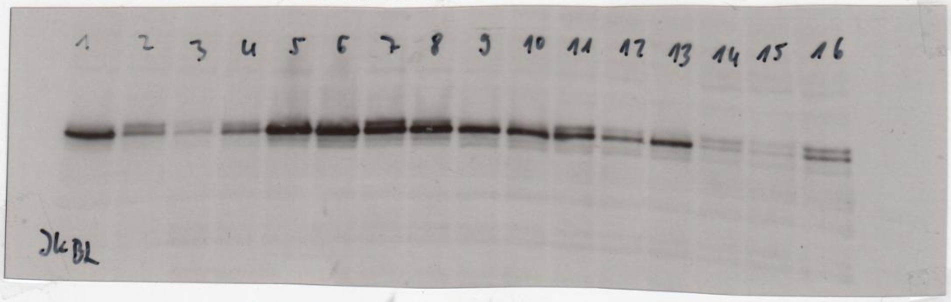

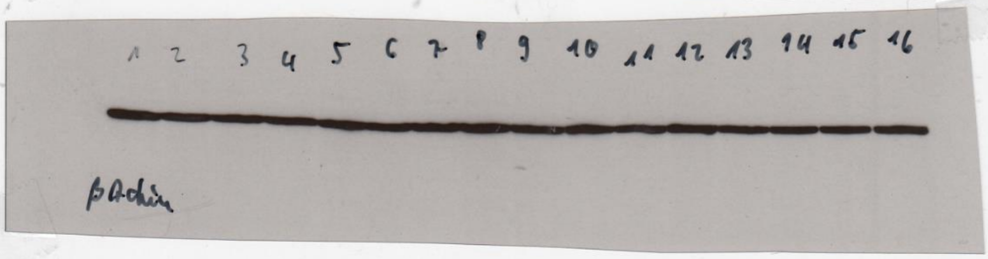

Exp. 508 **Abb. 4E**

1) HCT116PI3Kmut Kontrolle
2) HCT116PI3Kmut TNF 5 min
3) HCT116PI3Kmut TNF 15 min
4) HCT116PI3Kmut TNF 1 45 min
5) HCT116PI3Kmut -RIP1 k.o Kontrolle
6) HCT116PI3Kmut - RIP1 k.o TNF 5 min
7) HCT116PI3Kmut - RIP1 k.o TNF 15 min
8) HCT116PI3Kmut - RIP1 k.o TNF 45 min

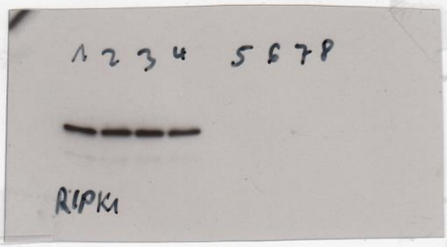

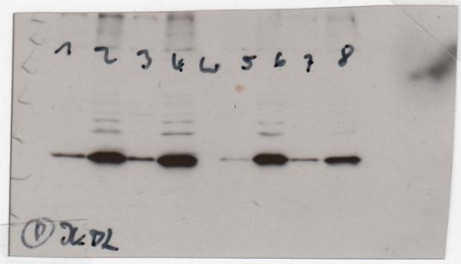

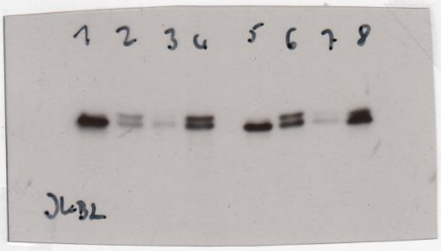

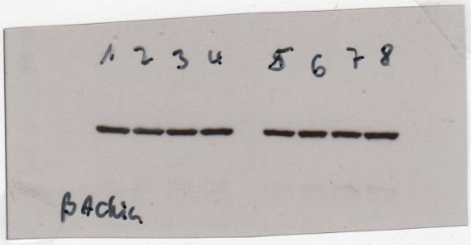

17

Exp. 507 Abb.4E

1) HeLaRIP3 Kontrolle
2) HeLaRIP3 TNF 5 min
3) HeLaRIP3 TNF 15 min
4) HeLaRIP3 TNF 1 45 min
5) HeLaRIP3-RIP1 k.o Kontrolle
6) HeLaRIP3- RIP1 k.o TNF 5 min
7) HeLaRIP3- RIP1 k.o TNF 15 min
8) HeLaRIP3- RIP1 k.o TNF 45min

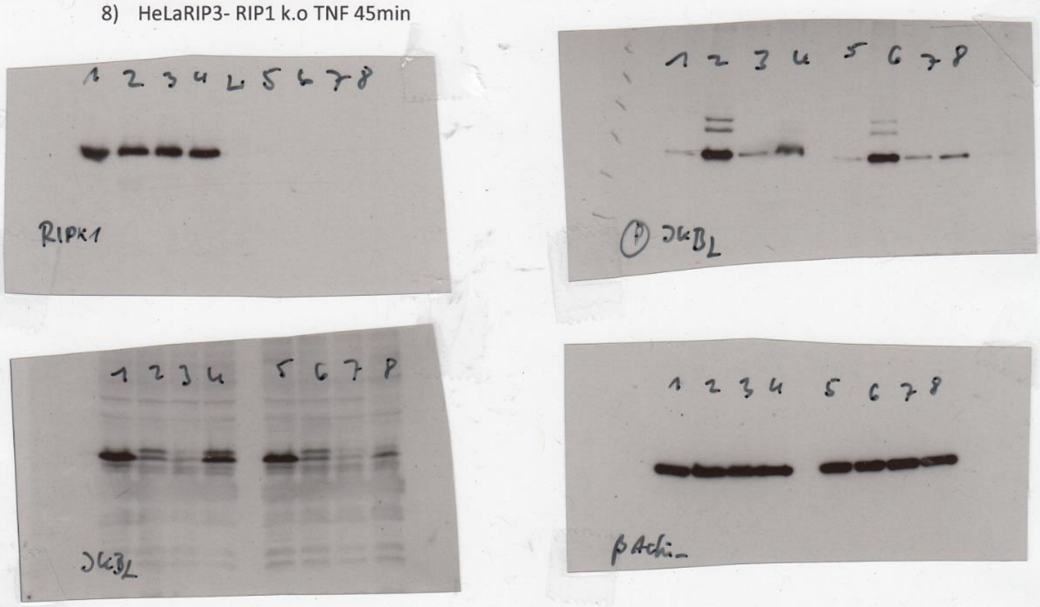

Exp. 506 **Abb. 4E**

1) HT29 Kontrolle
2) HT29 TNF 5 min
3) HT29 TNF 15 min
4) HT29 TNF 1 45 min
5) HT29-RIP1 k.o Kontrolle
6) HT29- RIP1 k.o TNF 5 min
7) HT29- RIP1 k.o TNF 15 min
8) HT29- RIP1 k.o TNF 45min

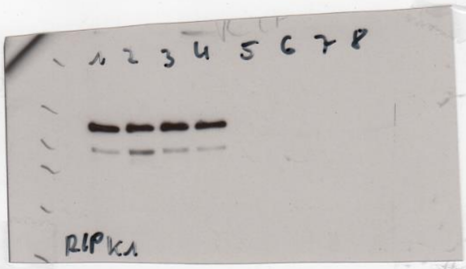

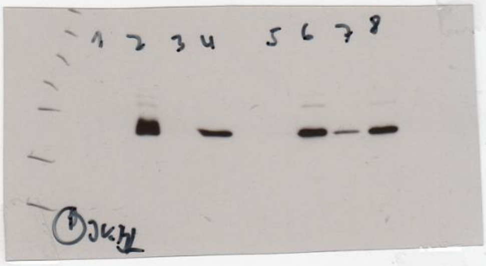

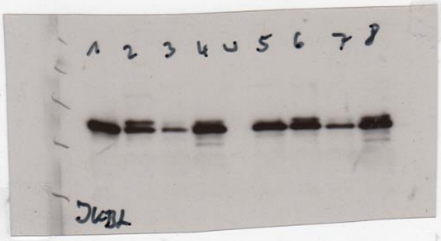

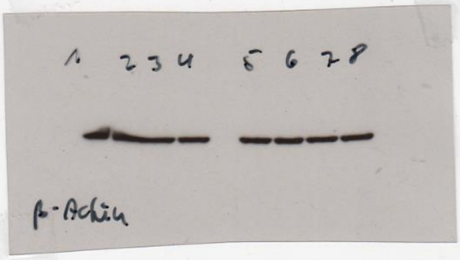

Exp. 327 **Abb. 5C**

1) HCT116PI3Kmut Kontrolle
2) HCT116PI3Kmut TNF 5 min
3) HCT116PI3Kmut TNF 15 min
4) HCT116PI3Kmut TNF 1 45 min
5) HCT116PI3Kmut-TRAF2 k.o Kontrolle
6) HCT116PI3Kmut-TRAF2 k.o TNF 5 min
7) HCT116PI3Kmut-TRAF2 k.o TNF 15 min
8) HCT116PI3Kmut-TRAF2 k.o TNF 45min
9) HCT116PI3Kmut-RIP k.o Kontrolle
10) HCT116PI3Kmut- RIP k.o TNF 5 min
11) HCT116PI3Kmut- RIP k.o TNF 15min
12) HCT116PI3Kmut- RIP k.o TNF 45 min
13) HCT116PI3Kmut-TRAF2 k.o –RIP k.o Kontrolle
14) HCT116PI3Kmut-TRAF2 k.o –RIP k.o TNF 5 min
15) HCT116PI3Kmut-TRAF2 k.o –RIP k.o TNF 15 min
16) HCT116PI3Kmut-TRAF2 k.o–RIP k.o TNF45 min

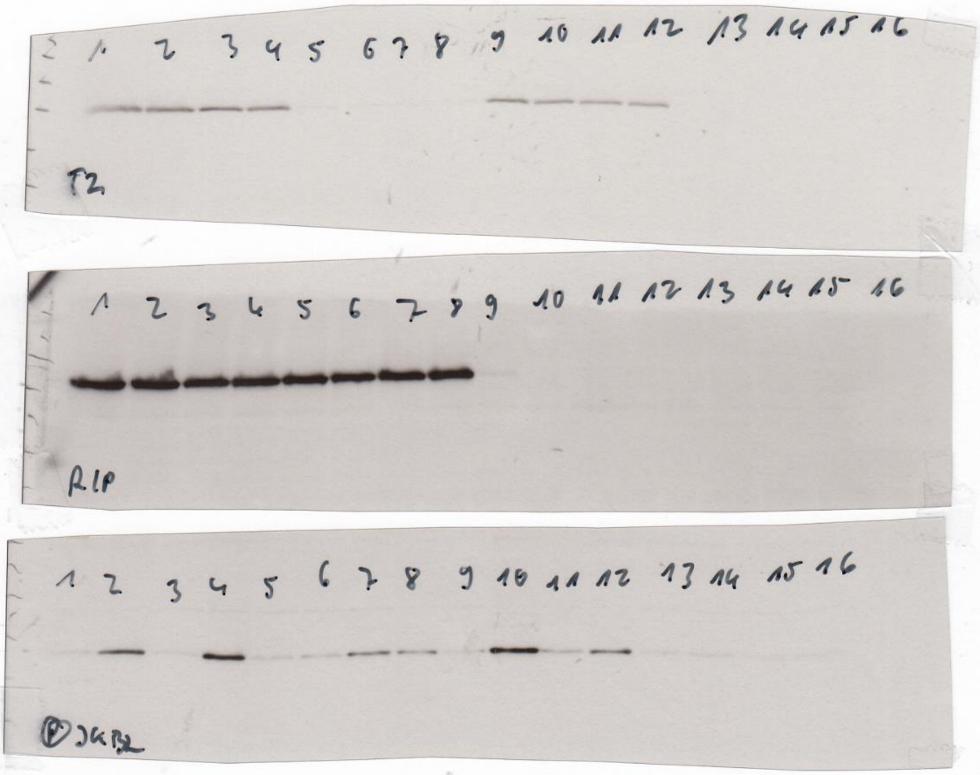

Exp. 327 **Abb. 5** C

1) HCT116PI3Kmut Kontrolle
2) HCT116PI3Kmut TNF 5 min
3) HCT116PI3Kmut TNF 15 min
4) HCT116PI3Kmut TNF 1 45 min
5) HCT116PI3Kmut-TRAF2 k.o Kontrolle
6) HCT116PI3Kmut-TRAF2 k.o TNF 5 min
7) HCT116PI3Kmut-TRAF2 k.o TNF 15 min
8) HCT116PI3Kmut-TRAF2 k.o TNF 45min
9) HCT116PI3Kmut-RIP k.o Kontrolle
10) HCT116PI3Kmut- RIP k.o TNF 5 min
11) HCT116PI3Kmut- RIP k.o TNF 15min
12) HCT116PI3Kmut- RIP k.o TNF 45 min
13) HCT116PI3Kmut-TRAF2 k.o –RIP k.o Kontrolle
14) HCT116PI3Kmut-TRAF2 k.o –RIP k.o TNF 5 min
15) HCT116PI3Kmut-TRAF2 k.o –RIP k.o TNF 15 min
16) HCT116PI3Kmut-TRAF2 k.o–RIP k.o TNF45 min

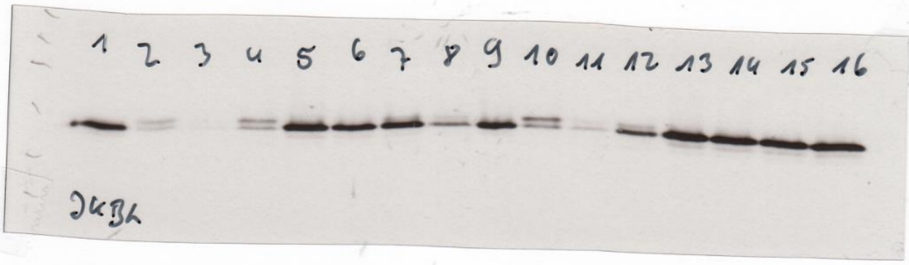

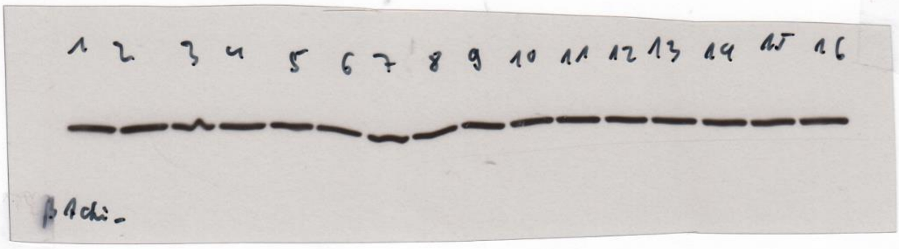

Exp. 529 **Abb. 5C**

1) HCT116PI3Kmut Kontrolle
2) HCT116PI3K mut TNF 5 min
3) HCT116PI3K mut TNF 15 min
4) HCT116PI3K mut TNF 1 45 min
5) HCT116PI3K mut -TRAF2 k.o Kontrolle
6) HCT116PI3K mut -TRAF2 k.o TNF 5 min
7) HCT116PI3K mut -TRAF2 k.o TNF 15 min
8) HCT116PI3K mut -TRAF2 k.o TNF 45min
9) HCT116PI3K mut +Degrader 1 µM 24 h
10) HCT116PI3K mut +Degrader 1 µM 24 h TNF 5 min
11) HCT116PI3K mut +Degrader 1 µM 24 h TNF 15 min
12) HCT116PI3K mut +Degrader 1 µM 24 h TNF 1 45 min
13) HCT116PI3K mut -TRAF2 k.o +Degrader 1 µM 24 h
14) HCT116PI3K mut -TRAF2 k.o +Degrader 1 µM 24 h TNF 5 min
15) HCT116PI3K mut -TRAF2 k.o +Degrader 1 µM 24 h TNF 15 min
16) HCT116PI3K mut -TRAF2 k.o +Degrader 1 µM 24 h TNF 45min

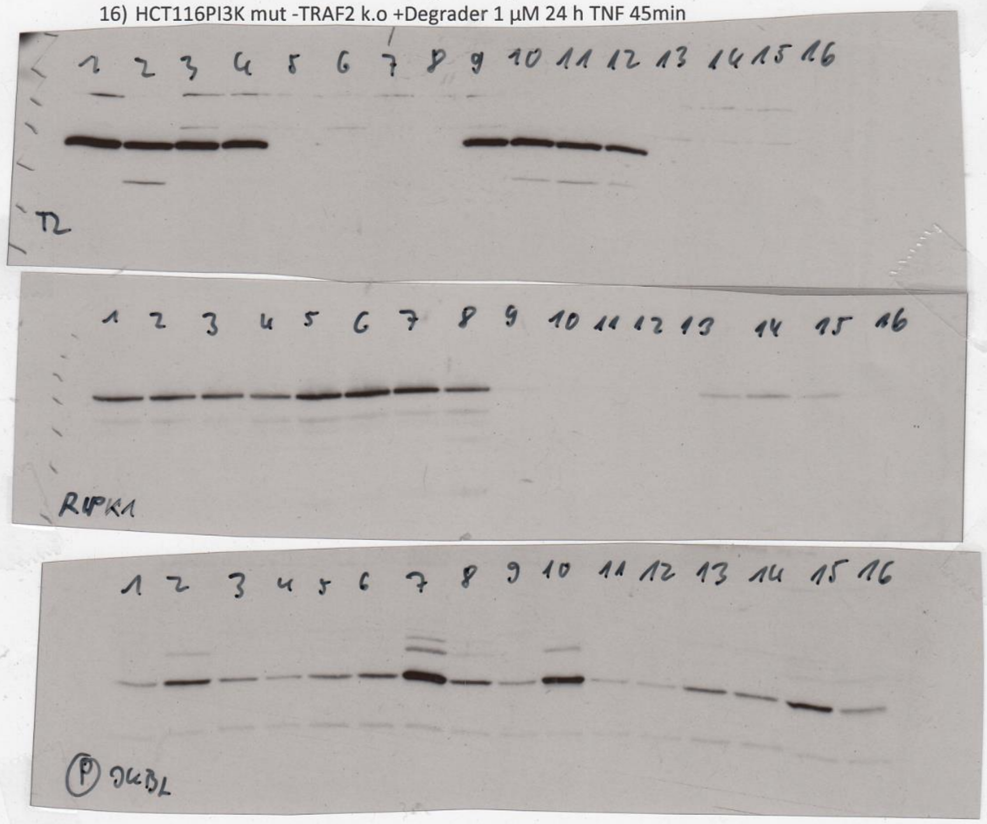

22

Exp. 529 **Abb. 5C**

1) HCT116PI3Kmut Kontrolle
2) HCT116PI3K mut TNF 5 min
3) HCT116PI3K mut TNF 15 min
4) HCT116PI3K mut TNF 1 45 min
5) HCT116PI3K mut -TRAF2 k.o Kontrolle
6) HCT116PI3K mut -TRAF2 k.o TNF 5 min
7) HCT116PI3K mut -TRAF2 k.o TNF 15 min
8) HCT116PI3K mut -TRAF2 k.o TNF 45min
9) HCT116PI3K mut +Degrader 1 µM 24 h
10) HCT116PI3K mut +Degrader 1 µM 24 h TNF 5 min
11) HCT116PI3K mut +Degrader 1 µM 24 h TNF 15 min
12) HCT116PI3K mut +Degrader 1 µM 24 h TNF 1 45 min
13) HCT116PI3K mut -TRAF2 k.o +Degrader 1 µM 24 h
14) HCT116PI3K mut -TRAF2 k.o +Degrader 1 µM 24 h TNF 5 min
15) HCT116PI3K mut -TRAF2 k.o +Degrader 1 µM 24 h TNF 15 min
16) HCT116PI3K mut -TRAF2 k.o +Degrader 1 µM 24 h TNF 45min

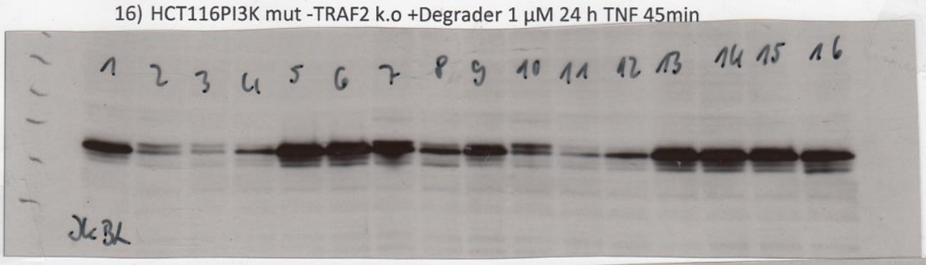

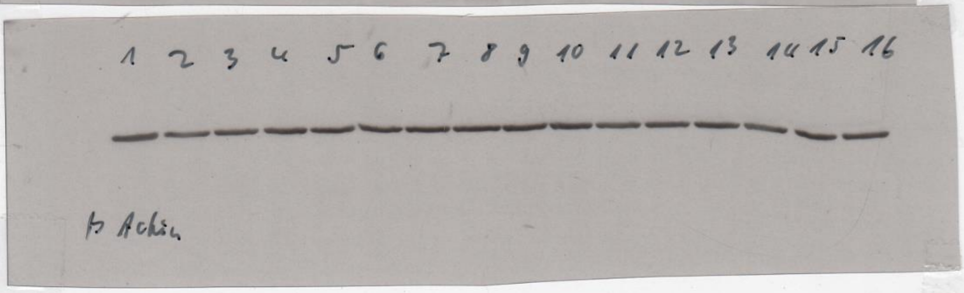

Exp. 500 **Abb. 5C**

1) HeLaRIP3 Kontrolle
2) HeLaRIP3 TNF 5 min
3) HeLaRIP3 TNF 15 min
4) HeLaRIP3 TNF 1 45 min
5) HeLaRIP3-TRAF2 k.o Kontrolle
6) HeLaRIP3-TRAF2 k.o TNF 5 min
7) HeLaRIP3-TRAF2 k.o TNF 15 min
8) HeLaRIP3-TRAF2 k.o TNF 45min
9) HeLaRIP3 +Degrader 1 µM 24 h
10) HeLaRIP3 +Degrader 1 µM 24 h TNF 5 min
11) HeLaRIP3 +Degrader 1 µM 24 h TNF 15 min
12) HeLaRIP3 +Degrader 1 µM 24 h TNF 1 45 min
13) HeLaRIP3-TRAF2 k.o +Degrader 1 µM 24 h
14) HeLaRIP3-TRAF2 k.o +Degrader 1 µM 24 h TNF 5 min
15) HeLaRIP3-TRAF2 k.o +Degrader 1 µM 24 h TNF 15 min
16) HeLaRIP3-TRAF2 k.o +Degrader 1 µM 24 h TNF 45min

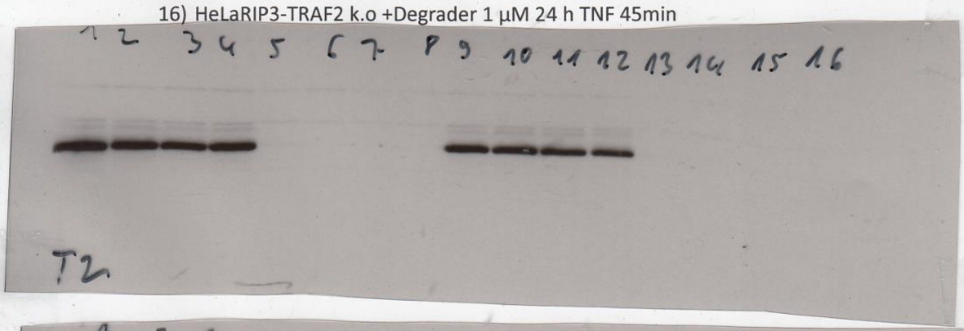

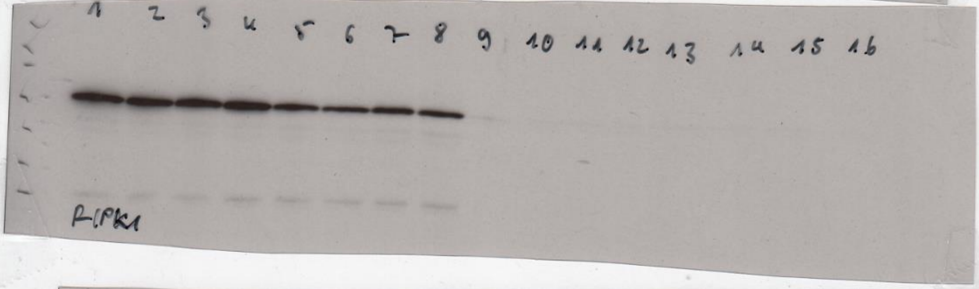

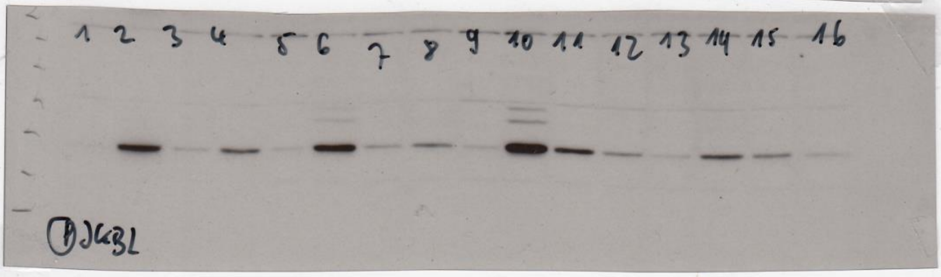

Exp. 500

Abb. 5C

1) HeLaRIP3 Kontrolle
2) HeLaRIP3 TNF 5 min
3) HeLaRIP3 TNF 15 min
4) HeLaRIP3 TNF 1 45 min
5) HeLaRIP3-TRAF2 k.o Kontrolle
6) HeLaRIP3-TRAF2 k.o TNF 5 min
7) HeLaRIP3-TRAF2 k.o TNF 15 min
8) HeLaRIP3-TRAF2 k.o TNF 45min
9) HeLaRIP3 +Degrader 1 µM 24 h
10) HeLaRIP3 +Degrader 1 µM 24 h TNF 5 min
11) HeLaRIP3 +Degrader 1 µM 24 h TNF 15 min
12) HeLaRIP3 +Degrader 1 µM 24 h TNF 1 45 min
13) HeLaRIP3-TRAF2 k.o +Degrader 1 µM 24 h
14) HeLaRIP3-TRAF2 k.o +Degrader 1 µM 24 h TNF 5 min
15) HeLaRIP3-TRAF2 k.o +Degrader 1 µM 24 h TNF 15 min
16) HeLaRIP3-TRAF2 k.o +Degrader 1 µM 24 h TNF 45min

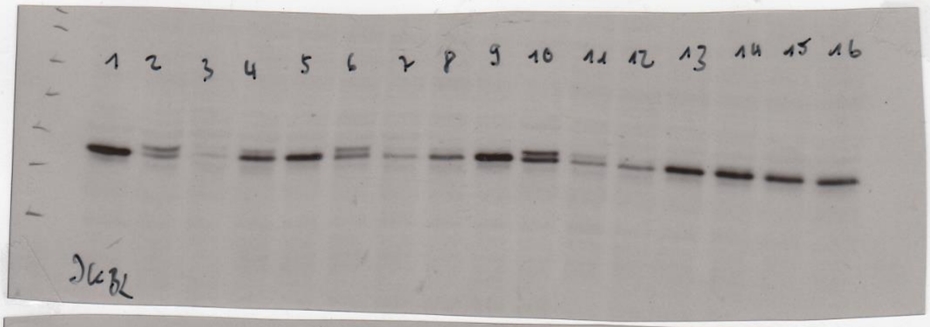

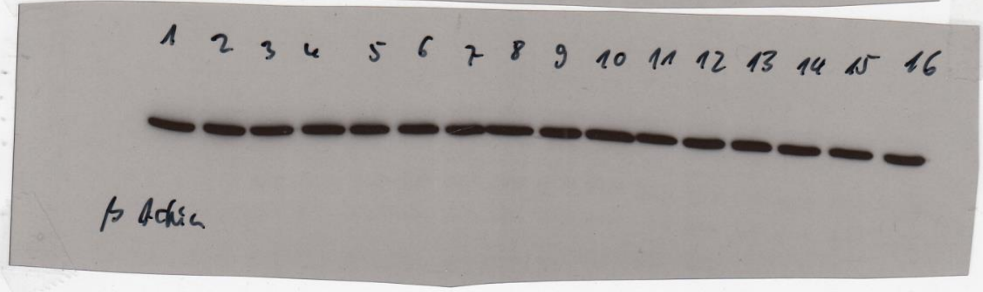

25

Exp. 503 **Abb. 5C**

1) HT29 Kontrolle
2) HT29 TNF 5 min
3) HT29 TNF 15 min
4) HT29 TNF 1 45 min
5) HT29-TRAF2 k.o Kontrolle
6) HT29-TRAF2 k.o TNF 5 min
7) HT29-TRAF2 k.o TNF 15 min
8) HT29-TRAF2 k.o TNF 45min
9) HT29 +Degrader 1 µM 24 h
10) HT29 +Degrader 1 µM 24 h TNF 5 min
11) HT29 +Degrader 1 µM 24 h TNF 15 min
12) HT29 +Degrader 1 µM 24 h TNF 1 45 min
13) HT29-TRAF2 k.o +Degrader 1 µM 24 h
14) HT29-TRAF2 k.o +Degrader 1 µM 24 h TNF 5 min
15) HT29-TRAF2 k.o +Degrader 1 µM 24 h TNF 15 min
16) HT29-TRAF2 k.o +Degrader 1 µM 24 h TNF 45min

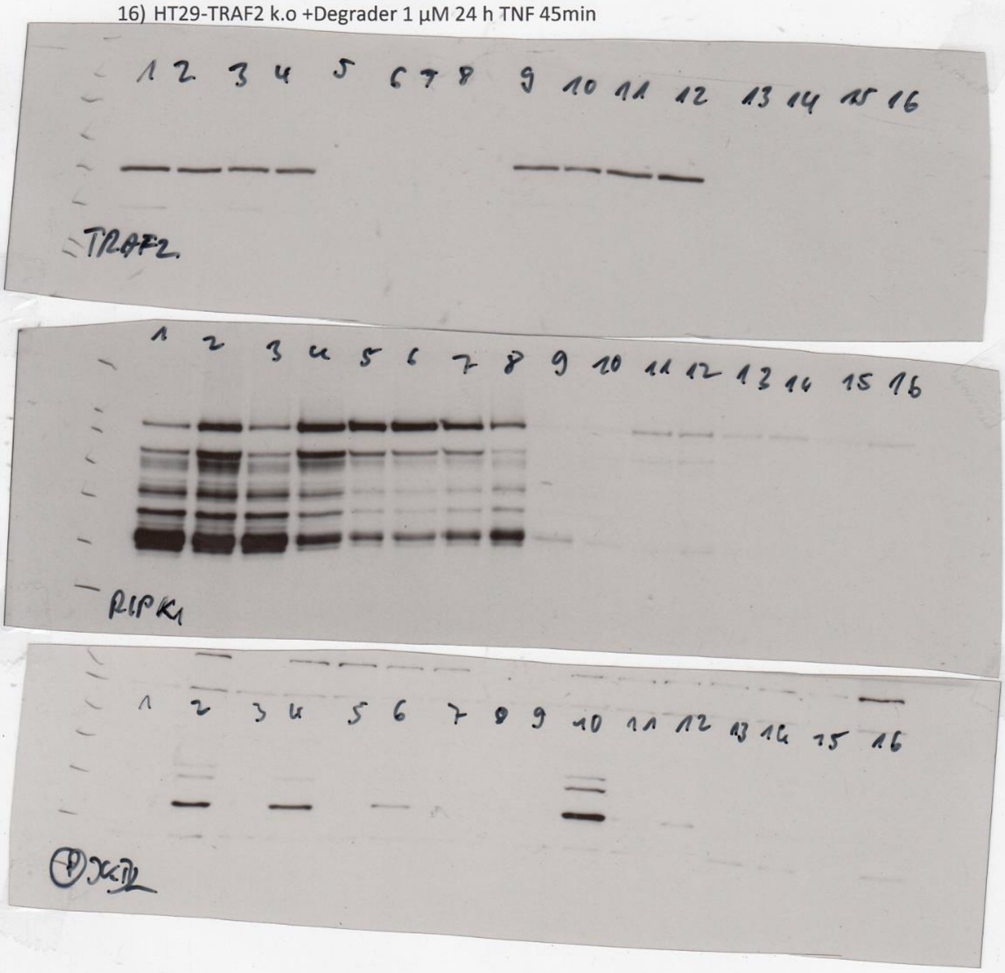

Exp. 503 **Abb. 5C**

1) HT29 Kontrolle
2) HT29 TNF 5 min
3) HT29 TNF 15 min
4) HT29 TNF 1 45 min
5) HT29-TRAF2 k.o Kontrolle
6) HT29-TRAF2 k.o TNF 5 min
7) HT29-TRAF2 k.o TNF 15 min
8) HT29-TRAF2 k.o TNF 45min
9) HT29 +Degrader 1 µM 24 h
10) HT29 +Degrader 1 µM 24 h TNF 5 min
11) HT29 +Degrader 1 µM 24 h TNF 15 min
12) HT29 +Degrader 1 µM 24 h TNF 1 45 min
13) HT29-TRAF2 k.o +Degrader 1 µM 24 h
14) HT29-TRAF2 k.o +Degrader 1 µM 24 h TNF 5 min
15) HT29-TRAF2 k.o +Degrader 1 µM 24 h TNF 15 min
16) HT29-TRAF2 k.o +Degrader 1 µM 24 h TNF 45min

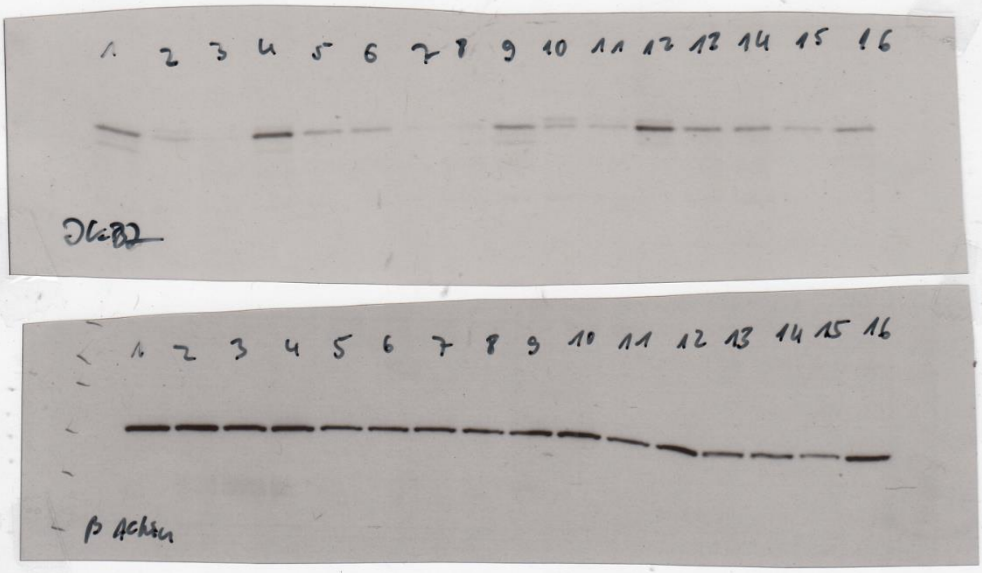

IP 44: 08.08.23 **Abb.7 (Lysat)**

1) HCT116PI3Kmut+5 ng TNF60-Fc
2) HCT116PI3Kmut+1 µg/ml TNF60-Fc 10 min
3) HCT116PI3Kmut-TRAF2 k.o+5 ng TNF60-Fc
4) HCT116PI3Kmut-TRAF2 k.o+1 µg/ml TNF60-Fc 10 min
5) HCT116PI3Kmut-RIP k.o+5 ng TNF60-Fc
6) HCT116PI3Kmut-RIP k.o+1 µg/ml TNF60-Fc 10 min
7) HCT116PI3Kmut-TRAF2 k.o-RIP k.o+5 ng TNF60-Fc
8) HCT116PI3Kmut-TRAF2 k.o-RIP k.o+1 µg/ml TNF60-Fc 10 min

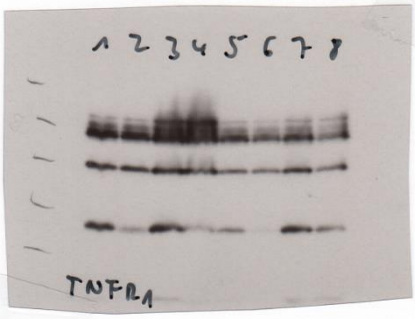

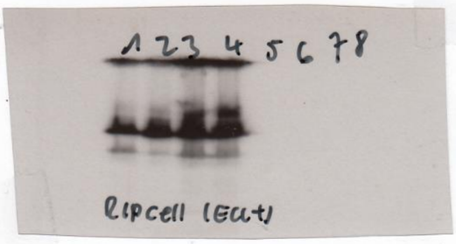

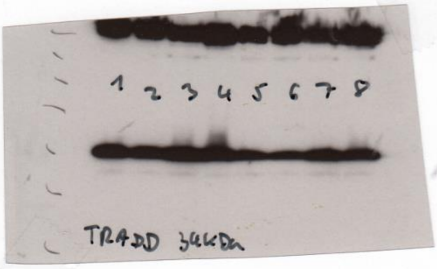

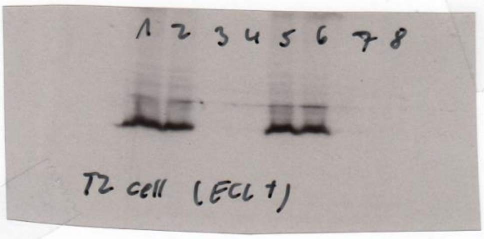

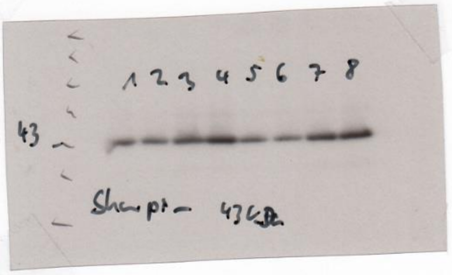

IP 44: 08.08.23 **Abb.7 (IP)**

1) HCT116PI3Kmut+5 ng TNF60-Fc
2) HCT116PI3Kmut+1 µg/ml TNF60-Fc 10 min
3) HCT116PI3Kmut-TRAF2 k.o+5 ng TNF60-Fc
4) HCT116PI3Kmut-TRAF2 k.o+1 µg/ml TNF60-Fc 10 min
5) HCT116PI3Kmut-RIP k.o+5 ng TNF60-Fc
6) HCT116PI3Kmut-RIP k.o+1 µg/ml TNF60-Fc 10 min
7) HCT116PI3Kmut-TRAF2 k.o-RIP k.o+5 ng TNF60-Fc
8) HCT116PI3Kmut-TRAF2 k.o-RIP k.o+1 µg/ml TNF60-Fc 10 min

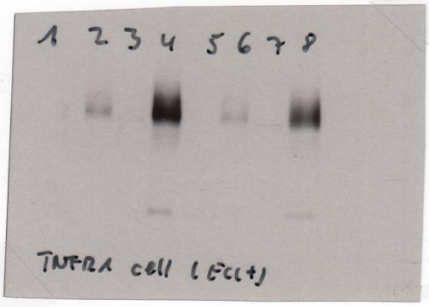

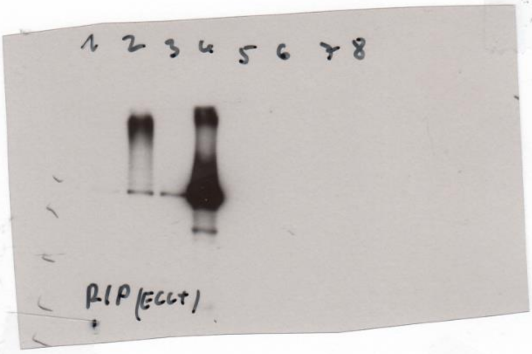

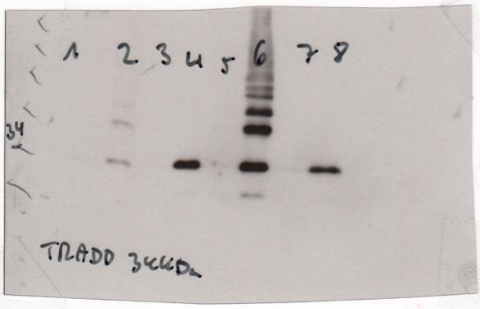

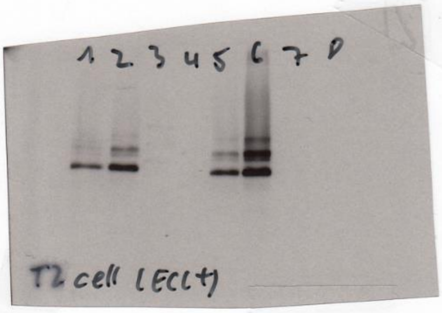

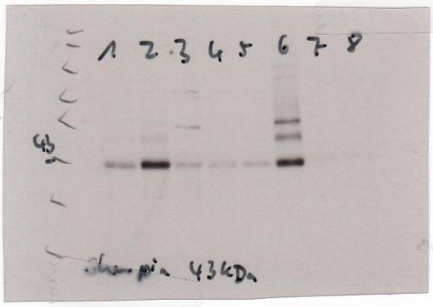

[illegible]

29

Abb7 (45)

9) HCT116PI3Kmut+5 ng FasL-Fc
10) HCT116PI3Kmut+1 µg/ml FasL-Fc 1 h
11) HCT116PI3Kmut-TRAF2 k.o+5 ng FasL-Fc
12) HCT116PI3Kmut-TRAF2 k.o+1 µg/ml FasL-Fc 1 h
13) HCT116PI3Kmut-RIP k.o+5 ng FasL-Fc
14) HCT116PI3Kmut-RIP k.o+1 µg/ml FasL-Fc 1 h
15) HCT116PI3Kmut-TRAF2 k.o-RIP k.o+5 ng FasL-Fc
16) HCT116PI3Kmut-TRAF2 k.o-RIP k.o+1 µg/ml FasL-Fc 1 h j

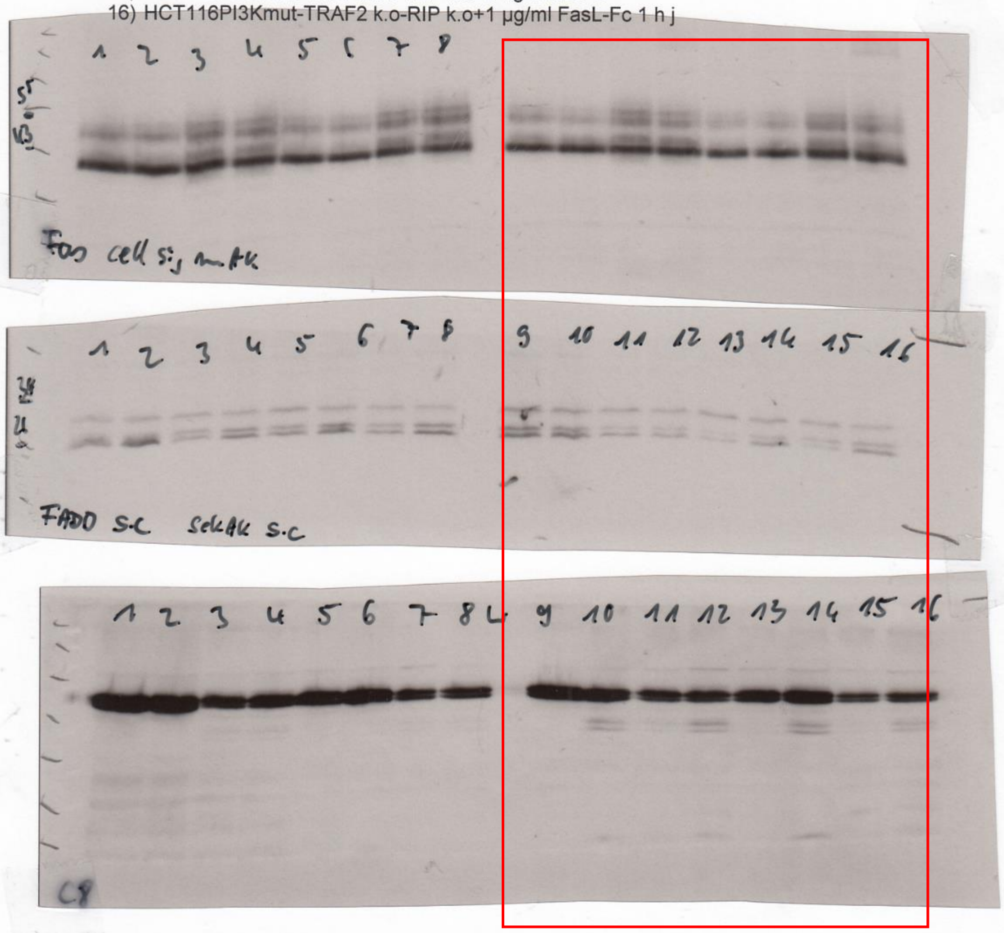

[illegible]

Abb.7 (Lys)

9) HCT116PI3Kmut+5 ng FasL-Fc
10) HCT116PI3Kmut+1 µg/ml FasL-Fc 1 h
11) HCT116PI3Kmut-TRAF2 k.o+5 ng FasL-Fc
12) HCT116PI3Kmut-TRAF2 k.o+1 µg/ml FasL-Fc 1 h
13) HCT116PI3Kmut-RIP k.o+5 ng FasL-Fc
14) HCT116PI3Kmut-RIP k.o+1 µg/ml FasL-Fc 1 h
15) HCT116PI3Kmut-TRAF2 k.o-RIP k.o+5 ng FasL-Fc
16) HCT116PI3Kmut-TRAF2 k.o-RIP k.o+1 µg/ml FasL-Fc 1 h j

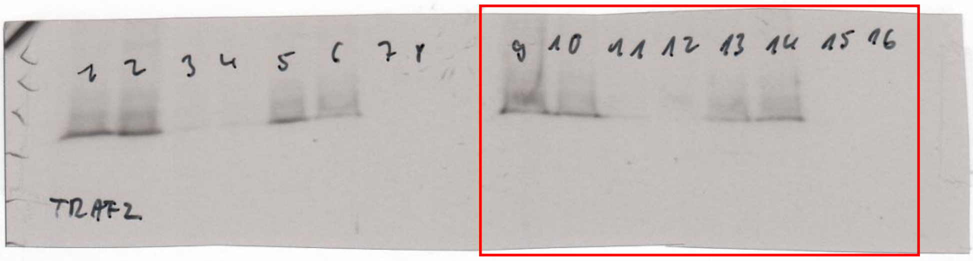

31

Abb7 (Lys)

9) HCT116PI3Kmut+5 ng FasL-Fc
10) HCT116PI3Kmut+1 µg/ml FasL-Fc 1 h
11) HCT116PI3Kmut-TRAF2 k.o+5 ng FasL-Fc
12) HCT116PI3Kmut-TRAF2 k.o+1 µg/ml FasL-Fc 1 h
13) HCT116PI3Kmut-RIP k.o+5 ng FasL-Fc
14) HCT116PI3Kmut-RIP k.o+1 µg/ml FasL-Fc 1 h
15) HCT116PI3Kmut-TRAF2 k.o-RIP k.o+5 ng FasL-Fc
16) HCT116PI3Kmut-TRAF2 k.o-RIP k.o+1 µg/ml FasL-Fc 1 h

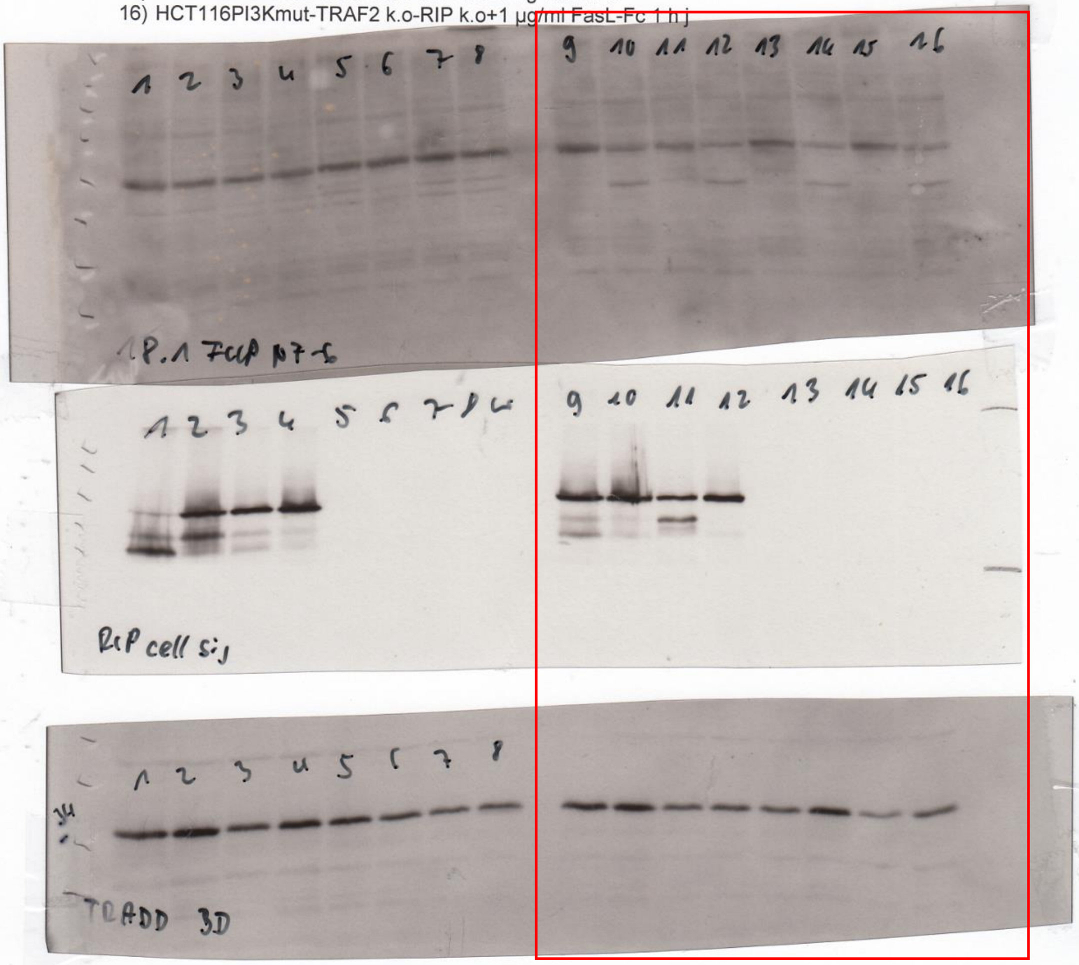

[illegible]

32

Abb. 7 (IP)

9) HCT116PI3Kmut+5 ng FasL-Fc
10) HCT116PI3Kmut+1 µg/ml FasL-Fc 1 h
11) HCT116PI3Kmut-TRAF2 k.o+5 ng FasL-Fc
12) HCT116PI3Kmut-TRAF2 k.o+1 µg/ml FasL-Fc 1 h
13) HCT116PI3Kmut-RIP k.o+5 ng FasL-Fc
14) HCT116PI3Kmut-RIP k.o+1 µg/ml FasL-Fc 1 h
15) HCT116PI3Kmut-TRAF2 k.o-RIP k.o+5 ng FasL-Fc
16) HCT116PI3Kmut-TRAF2 k.o-RIP k.o+1 µg/ml FasL-Fc 1 h j

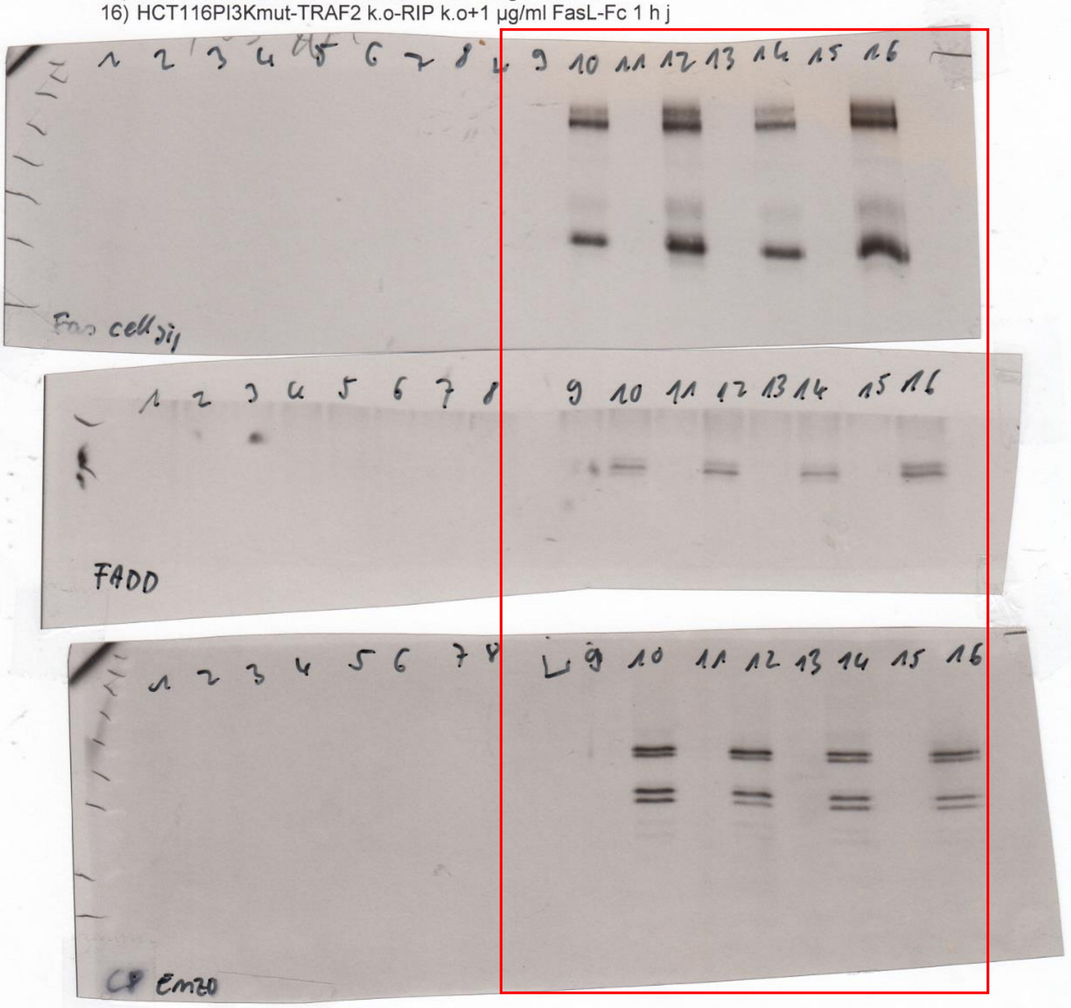

33

Abb.7 (IP)

9) HCT116PI3Kmut+5 ng FasL-Fc
10) HCT116PI3Kmut+1 µg/ml FasL-Fc 1 h
11) HCT116PI3Kmut-TRAF2 k.o+5 ng FasL-Fc
12) HCT116PI3Kmut-TRAF2 k.o+1 µg/ml FasL-Fc 1 h
13) HCT116PI3Kmut-RIP k.o+5 ng FasL-Fc
14) HCT116PI3Kmut-RIP k.o+1 µg/ml FasL-Fc 1 h
15) HCT116PI3Kmut-TRAF2 k.o-RIP k.o+5 ng FasL-Fc
16) HCT116PI3Kmut-TRAF2 k.o-RIP k.o+1 µg/ml FasL-Fc 1 h j

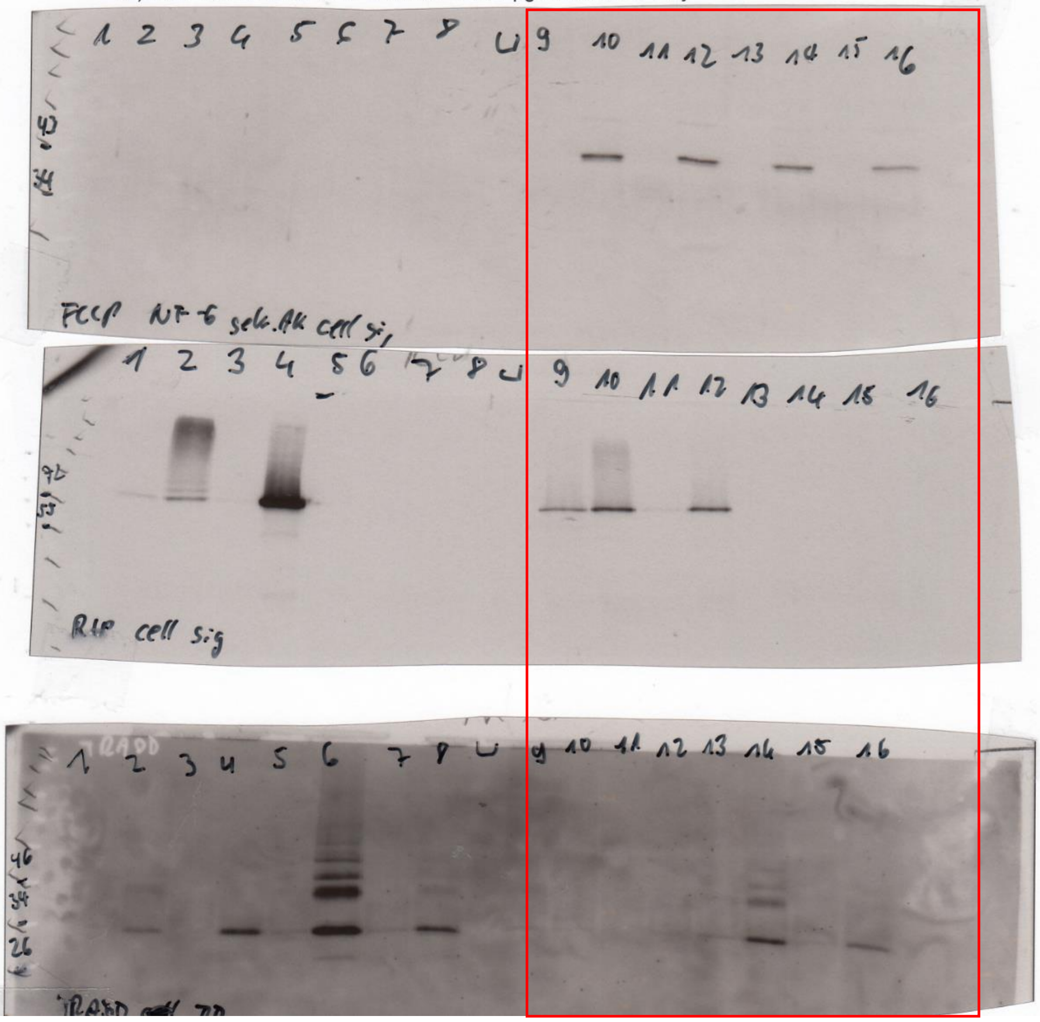

Abb. 7 (IP)

9) HCT116PI3Kmut+5 ng FasL-Fc
10) HCT116PI3Kmut+1 µg/ml FasL-Fc 1 h
11) HCT116PI3Kmut-TRAF2 k.o+5 ng FasL-Fc
12) HCT116PI3Kmut-TRAF2 k.o+1 µg/ml FasL-Fc 1 h
13) HCT116PI3Kmut-RIP k.o+5 ng FasL-Fc
14) HCT116PI3Kmut-RIP k.o+1 µg/ml FasL-Fc 1 h
15) HCT116PI3Kmut-TRAF2 k.o-RIP k.o+5 ng FasL-Fc
16) HCT116PI3Kmut-TRAF2 k.o-RIP k.o+1 µg/ml FasL-Fc 1 h j

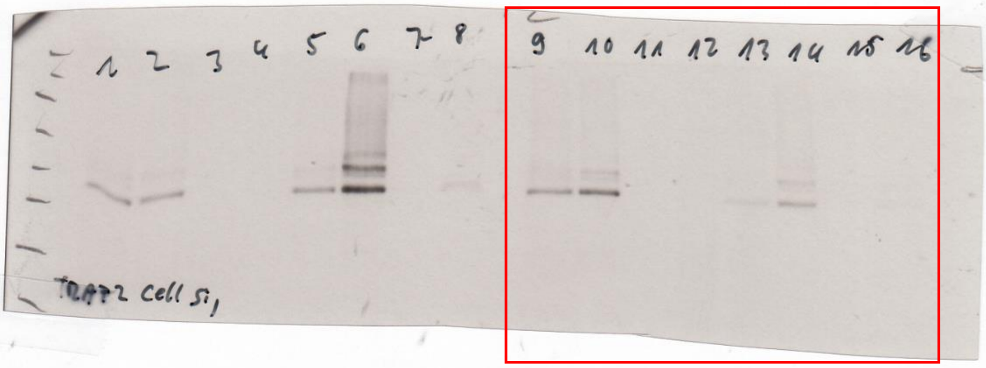

Supplement: Supplementary file 2 — supplemental data original WBs [file 41419_2024_7325_MOESM2_ESM.pdf]
